# Plasmonic sensors based on graphene and graphene hybrid materials

**DOI:** 10.1186/s40580-022-00319-5

**Published:** 2022-06-13

**Authors:** Zhichao Zhang, Yeageun Lee, Md Farhadul Haque, Juyoung Leem, Ezekiel Y. Hsieh, SungWoo Nam

**Affiliations:** 1 Department of Mechanical Science and Engineering, University of Illinois at Urbana-Champaign, Urbana, IL 61801 USA; 2 Department of Mechanical Engineering, Stanford University, Stanford, CA 94305 USA; 3 TomKat Center for Sustainable Energy, Stanford University, Stanford, CA 94305 USA; 4 Department of Mechanical and Aerospace Engineering, University of California Irvine, Irvine, CA 92697 USA

**Keywords:** Graphene, Plasmonics, Graphene-hybrids, Plasmonic sensors, Biosensors, Optical sensors, Chemical sensors

## Abstract

The past decade has witnessed a rapid growth of graphene plasmonics and their applications in different fields. Compared with conventional plasmonic materials, graphene enables highly confined plasmons with much longer lifetimes. Moreover, graphene plasmons work in an extended wavelength range, i.e., mid-infrared and terahertz regime, overlapping with the fingerprints of most organic and biomolecules, and have broadened their applications towards plasmonic biological and chemical sensors. In this review, we discuss intrinsic plasmonic properties of graphene and strategies both for tuning graphene plasmons as well as achieving higher performance by integrating graphene with plasmonic nanostructures. Next, we survey applications of graphene and graphene-hybrid materials in biosensors, chemical sensors, optical sensors, and sensors in other fields. Lastly, we conclude this review by providing a brief outlook and challenges of the field. Through this review, we aim to provide an overall picture of graphene plasmonic sensing and to suggest future trends of development of graphene plasmonics.

## Introduction

Surface plasmons are collective electron oscillations confined at surfaces due to strong interactions with photons. Although surface plasmons have been increasingly studied by scientists in recent decades, they were in use even centuries ago for making beautiful Gothic stained glass adorning medieval cathedrals [1, 2]. These colors were produced by dispersing metal nanoparticles (NPs) into the glass resulting in surface plasmon excitations at the interfaces. Researchers have since discovered that surface plasmons can occur at certain wavelength ranges at the interfaces between metals with positive real part of relative permittivity and materials with negative real part of relative permittivity at certain range of wavelength [3]. Within the visible range specifically, surface plasmons can be formed at the interfaces between metals such as Au or Ag and dielectric layers such as air or silicon dioxide [4–6].

Graphene is a two-dimensional (2D) material with a 2D crystalline structure and atomically thin thickness. It exhibits extremely high mechanical strength and electrical mobility, is a semi-metal with zero bandgap, and can be easily sculpted into different structures such as nanoribbons or nanodisks with nanofabrication techniques. Due to its unique Dirac cone band structure with a linear dispersion relation, graphene shows extraordinary optical properties when used as a plasmonic material. In particular, graphene can localize electromagnetic fields within the small vicinity of graphene sheet, and its spectral features can be easily tuned by external electrostatic gating or chemical doping [7–9]. For this reason, graphene is an interesting candidate for probing small changes in surrounding environments at micro- and nano-scales, and this constitutes the fundamental working mechanisms behind plasmonic sensors. Additionally, graphene surface plasmons are within the mid-infrared (mid-IR) and terahertz (THz) range, tremendously extending their applications for sensing organic and biomolecules which cannot be easily achieved with conventional plasmonic materials.

Graphene plasmonics is a new and rapidly growing research area, with researchers only having started to fill the vacancy of experimental research on graphene plasmons in the past decade. In 2011, Ju et al. experimentally demonstrated that graphene plasmons can be induced by periodic graphene micro-ribbons, and the resonance can be tuned via ribbon width and electrostatic doping [10]. Since these discoveries, the field has witnessed a sharp increase in graphene plasmonic sensors in different fields, including biosensors [7, 11, 12], chemical sensors [13–16], photodetectors [10, 17–21], and more. A number of review papers related to graphene plasmonics have already been published due to growing interest in this field. However, these have focused only on the theoretical background [22–25], or specific configurations and applications [26–29]. In this review, in addition to discussing the background physics, we systematically summarize the approaches that have been adopted for exciting and adjusting plasmons in both graphene and graphene-based hybrid structures and present the most up-to-date sensing applications for both types of plasmonic structures.

Following our introductory section, in Sect. 2, we will discuss the intrinsic plasmonic properties of graphene and approaches that can be applied for tuning graphene plasmons. In Sect. 3, we will introduce plasmonic effects in graphene-based hybrid materials. We will discuss the roles of graphene in different systems and the effects of different types of metal nanostructures. In Sect. 4, we will review sensing applications of graphene as well as graphene-based hybrid materials. We will primarily focus on three major types of sensors which have been widely investigated, namely biosensors, chemical sensors, and optical sensors, followed by a brief discussion of sensors that do not fall into these three categories. Finally, we will conclude with a brief outlook. The overall goal of this review is to comprehensively discuss the current state-of-the-art in graphene plasmon excitation techniques and provide readers with ideas for nanophotonic and plasmonic sensing.

## Intrinsic plasmonic properties of graphene

Plasmons are the collective oscillations of electrons at the surface of a metal or metal-dielectric interface subjected to an external electromagnetic field. The plasmonic oscillations at the material surface or interface create confined electromagnetic fields, called surface plasmon polaritons (SPP). SPPs are common phenomena often found in metal or semi-metal surfaces such as graphene [30, 31]. Compared with conventional noble metal plasmons, graphene plasmons have three distinctive advantages [25, 32]. First, graphene has an exceptionally high electron mobility, and graphene plasmons therefore exhibit low loss. Second, graphene plasmons are highly confined within a small spatial region since the plasmonic wavelength can be two orders of magnitude smaller than the incident light. Third, graphene plasmon wavelength is widely tunable through structural modulation and/or electrostatic doping. These advantages make graphene a promising candidate for plasmonic applications such as sensing, light-harvesting, or spectroscopy [11].

In this section, we will discuss popular ways that have been applied for exciting graphene plasmons, such as introducing edges via micro/nano patterning, introducing and controlling defects, selective doping, and spatial modulations of electric potential.

### Graphene plasmons in micro/nano patterned structures

Introducing edges on graphene is an effective way for confining SPPs and inducing plasmon resonance [30–33]. The plasmon frequency shifts from THz to mid-IR when the graphene structure is reduced from the microscale to 100 nm scale range. In order to probe graphene SPPs at these small length scales, atomic force microscopy (AFM) coupled with a light illumination setup is used. Specifically, AFM-IR uses IR-range illumination focused on a metallic AFM tip to launch SPPs on graphene. The propagating SPPs are reflected from the graphene edges and create standing waves between the tip and edge when interacting with the incoming SPPs.

Chen et al. experimentally demonstrated that incident light with a wavelength of 9.7 μm excited graphene plasmons when the width of patterned graphene was reduced to ~ 260 nm [32]. The near-field image revealed fringe patterns due to the standing wave. The distance between two maxima in the fringe pattern denoted twice the plasmonic wavelength. The plasmon wavelength reported in this study was 1/40th of the wavelength of the incident light, thereby showing strong light confinement of graphene. The reduced plasmonic wavelength was possible due to the 2D system and graphene’s unique conductance properties. Xu et al. reported that the width of the graphene nanoribbons plays a significant role in capturing localized plasmon resonance [31]. Near-field optical images revealed two localized modes when the width of the nanoribbon was smaller than the separation of the lowest-order modes of graphene plasmons. Depending on the ribbon width and the excitation wavelength, the localized modes were observed differently in the near-field images due to the constructive or destructive interferences between plasmonic wave and reflected waves at the edge of the graphene nanoribbon.

**Fig. 1:**
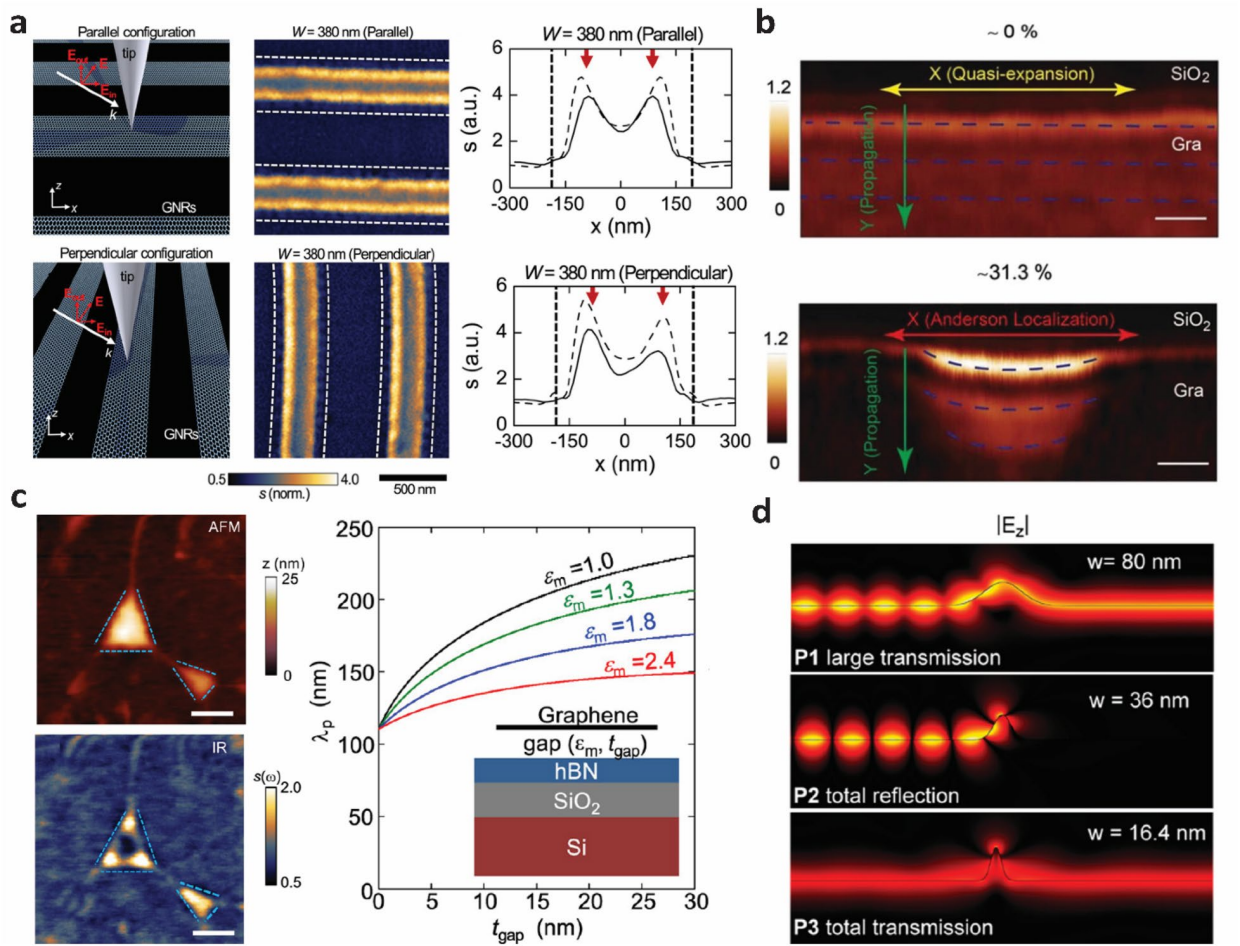
Graphene plasmons in graphene nanoribbons (**a**) and graphene with structural defects (**b**–**d**). **a** AFM-IR imaging with two different laser excitation configurations (left) and corresponding AFM-IR images of graphene nanoribbons at each configuration showing the orientation dependent near-field intensity. Schematics of the AFM-IR imaging (left), near-field images (middle) and line profile of the near-field images (right) when the E-field of the excitation was parallel (top) and perpendicular (bottom). The ribbon edge and fringe locations are denoted by the dashed line and red arrows on the line profiles. Laser excitation frequency was 1184 cm^− 1^. Reproduced with permission from [30]. Copyright 2017 American Chemical Society. **b** Near-field images of ordered or quasi-expansion (top) and highly disordered or Anderson localized (bottom) graphene at 901 cm^− 1^ excitation comparing the plasmonic confinement between the two graphene edges. The blue dashed lines represent the fringe pattern. Scale bar is 300 nm. Reproduced under the terms of the Creative Commons License from [34]. Copyright 2019 The Authors. Published by WILEY-VCH Verlag GmbH & Co. KGaA, Weinheim. **c** AFM topography (top left) and nano-IR image (bottom left) of graphene nanobubbles at 910 cm^− 1^ excitation showing plasmonic confinement at the bubbled regions. The boundaries of the nanobubbles are shown in blue dashed line in the nano-IR image. Scale bar is 200 nm. Right panel shows the theoretical calculation of plasmon wavelength and dispersion diagram of the graphene nanobubble structure. The bubble area is represented by the gap shown the inset schematics. Reproduced with permission from [35]. Copyright 2016 American Chemical Society. **d** Interaction of plasmon wave with a graphene wrinkle with three different widths (W). Reprinted with permission from [36]. Copyright 2017 American Chemical Society

Yan et al. demonstrated polarization dependent plasmonic resonance in graphene nanoribbons using a broadband excitation [33]. Localized plasmons in graphene nanoribbons were excited when the incident light was perpendicular to the nanoribbon length. When the light was parallel to the nanoribbon length, the light-matter interactions were weakened due to Pauli blocking of the interband transition. The localized plasmon resonance modes were characterized by multiple peaks in the extinction spectra of the nanoribbon structure. Hu et al. reported symmetric and asymmetric plasmonic interference fringes in AFM-IR images using a polarized laser at a 1184 cm^− 1^ excitation frequency (Fig. 1a) [30]. When the electric field (E-field) of the incident laser was parallel to the nanoribbon length, only graphene SPPs were activated. The corresponding fringe patterns in the near-field image were symmetric across the ribbon width (Fig. 1a, top). However, when the E-field was perpendicular to the ribbon length, surface plasmon resonance (SPR) was activated and interacted with the SPPs. The corresponding fringes showed asymmetric patterns across the ribbon width (Fig. 1a, bottom), with the asymmetricity attributed to coupling between SPR and SPP. In this case, the wider ribbon exhibited higher near-field intensity at the left fringe while the narrower ribbon exhibited higher near-field intensity at the right fringe. The spatial shift of the intensity enhancement was associated with different SPR modes. More specifically, for the wider nanoribbon, out-of-plane E-field (E_z_) direction was both downwards and upwards at either side of the ribbon. On the contrary, the E_z_ direction on the narrower ribbon was upwards on the right edge and downwards on the left edge, thereby inducing different SPR modes.

### Interaction of graphene plasmons with defects

Graphene plasmons can interact with defects, causing inhomogeneities in the density of states [34]. For example, defects or localized tensile strains in the graphene lattice can create disorder in the material system leading to Anderson localization of plasmon-polaritons. Duan et al. reported that relatively highly disordered (Anderson localized) graphene exhibited finite confinement under near-field imaging (Fig. 1b). The localization lengths along the graphene edge of disordered and ordered (i.e., defect-free) graphene samples were 247.3 nm and infinity, respectively, and the localization lengths were independent of the incident light frequency. Although the plasmonic wavelengths were similar in both ordered systems (216.7 nm) and disordered systems (220 nm) owing to identical graphene doping levels, the highly disordered systems exhibited stronger near-field confinement. The stronger field confinement observed in the disordered graphene can be explained by the scattering behavior of plasmons. In ordered systems, the plasmonic mean-free-path is much longer than its wavelength (1 μm and 200 nm, respectively), implying minimal scattering in plasmon transport. However, in highly disordered systems, plasmonic mean-free-path is much shorter (e.g., 44.1 nm), leading to a higher scattering strength.

Graphene plasmons also interact with out-of-plane topographies of graphene, such as bubbles, blisters, or wrinkles [35–37]. Such structural defects are often generated during transfer of 2D van der Waals (vdW) materials from one substrate to another. Fei et al. reported plasmonic hotspots in graphene-hexagonal boron nitride (hBN) heterostructure bubbles with ultra-high confinement (Fig. 1c) [35]. In this case, the hotspots occurred mainly due to the change in dielectric environments at the interface between the suspended graphene and graphene supported by the substrate. The reduction of the dielectric constant due to the distance between the substrate and the suspended graphene in the bubble increased the local plasmon wavelength. Increasing the distance further reduced the dielectric constant and further increased the plasmon wavelength. Another study suggested a different mechanism for IR absorption enhancement in hBN-graphene-hBN bubbles [37]. The higher strain in the bubbles induced doping in the graphene with energy higher than the Pauli Blocking, and the graphene doped beyond the Pauli threshold supported SPPs. The origin of the enhanced absorption was attributed to graphene SPPs at nanoscale strain domains in the bubbles.

Moreover, out-of-plane deformation can influence the propagation behavior of graphene plasmons. Slipchenko et al. reported computational studies on the behavior of plasmon propagation across a corrugation in graphene (Fig. 1d) [36]. For smooth corrugations (width > > height of the corrugation), the plasmon scattering depended on the dielectric environment. On the other hand, for sharp corrugations (width < height of the corrugation), the plasmon propagation instead depended on the aspect ratio of the corrugation. Specifically, when the width of the corrugation was wider than half of the plasmon wavelength, partial reflection occurred on the incident side of the corrugation. As the corrugation width was further reduced, the partial reflection became a total reflection with minimal transmission. Continued reduction of the corrugation width suggested total transmission for sharp corrugations in graphene.

### Investigating graphene plasmon by selective doping

**Fig. 2:**
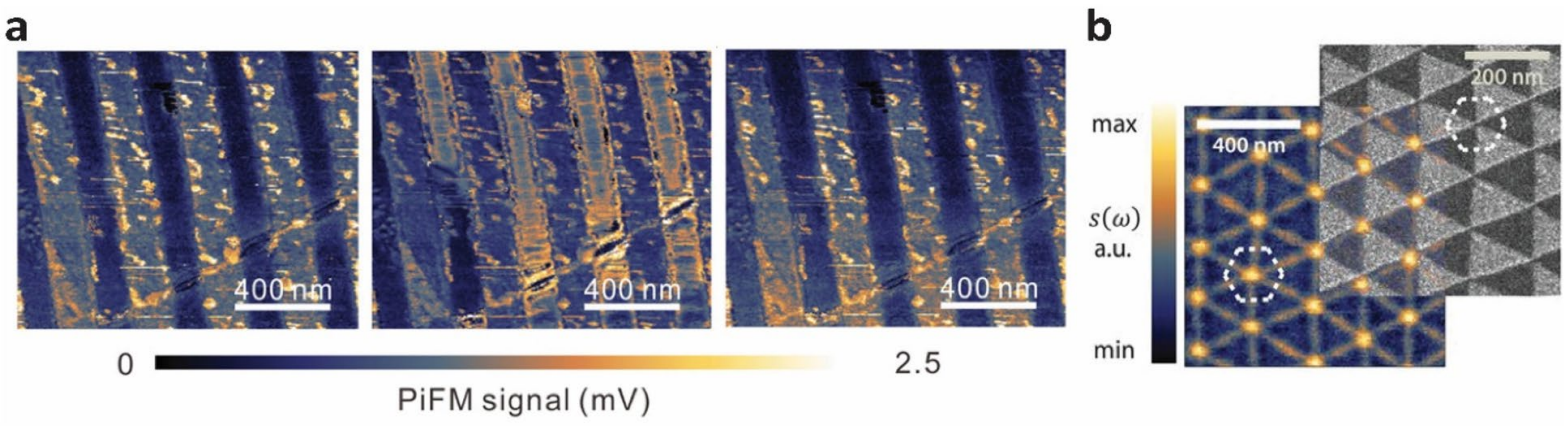
Interaction of graphene plasmons with carrier concentrations and Moiré superlattice. **a** Near-field images of graphene under selective doping by a patterned substrate at 1380 cm^− 1^ (left), 1496 cm^− 1^ (middle), and 1591 cm^− 1^ (right) excitation conditions. Reprinted with permission from [39]. Copyright 2018 WILEY-VCH Verlag GmbH & Co. KGaA, Weinheim. **b** Near-field image (left) of a twisted bilayer graphene overlapped with dark field TEM image (right). The dashed hexagon denotes unit cell of the light-matter interaction pattern. From [40], Reprinted with permission from AAAS

Plasmon wavelengths of graphene can be modulated by selective doping [38, 39]. Selective doping of graphene can be achieved in a variety of ways, and here we discuss selective doping via electrostatic gating [38] and transfer of graphene onto a substrate with patterns [39]. Alonso-Gonzalez et al. demonstrated the imaging of THz plasmons in a graphene photodetector with split-gates for the spatial control of carrier concentrations to generate a sharp p-n junction [38]. Under illumination with a metallic AFM tip, graphene plasmons were launched and produced oscillations of electric field intensity and local energy dissipation. The dissipated energy then heated up the p-n junction created by the split-gates and induced photocurrent via the photothermal effect. The photocurrent oscillated with a period of half the plasmon wavelength. The oscillation showed a linear correlation with the incident frequency, indicating acoustic THz plasmons. Liu et al. demonstrated selective doping of graphene using patterned dielectric-Au substrates and investigated graphene plasmons with this selective doping [39]. The substrate consisted of periodic structures of ridges and trenches with the transferred graphene supported by the ridges and suspended over the trenches. The plasmon wavelengths on the ridge and trench areas differed due to different doping levels induced by the substrate and air, respectively (Fig. 2a). As a result, plasmon confinement was observed at the ridge areas (supported graphene) with an incident wavenumber of 1380 cm^− 1^ (Fig. 2a, left panel). When the incident wavenumber was increased to 1496 cm^− 1^, plasmon confinement was observed at the trench areas (suspended graphene) (Fig. 2a, center panel). When the incident wavenumber was further increased to 1591 cm^− 1^, plasmon confinement returned to the ridge areas (Fig. 2a, right panel).

### Interaction of graphene plasmon in Moiré superlattice

Moiré superlattices formed by twisted bilayer graphene offer spatial modulation of the density of states, thereby providing an additional pathway for plasmon interactions in graphene [40–42]. Near-field images of twisted bilayer graphene revealed a Moiré pattern with a stronger near-field signal along the domain walls (Fig. 2b) [40]. A perpendicular electric displacement field (perpendicular to the graphene surface) opened the band gap in the AB or BA domains, thereby enhancing the optical conductivity at the domain walls. Jiang et al. used the reflection of SPPs from the soliton/domain wall of bilayer graphene to study tensile-type and shear-type soliton walls [41]. In twisted bilayer graphene, stacking order AB can shift towards BA stacking via a slight shift of the top layer with respect to the bottom layer, thereby creating tensile or shear strain induced one-dimensional (1D) soliton-like domain walls. Solitons parallel to the dislocation vector induced shear strain at the domain walls while solitons perpendicular to the dislocation direction induced tensile strain. The plasmons reflected off the shear or tensile domain-walls, and the reflected plasmons were observed as bright features under near field imaging. In their system, three distinct patterns in the near-field images were observed—triangles, L bends, and ovals. The shear solitons showed one bright line (triangle and L bend), while the tensile solitons showed two bright lines. Ni et al. reported the interaction of graphene SPPs with Moiré superlattices of graphene-hBN heterostructure [42]. In these heterostructures, the interface between plain graphene and Moiré patterned graphene acted as a plasmon reflector with doping dependent properties.

### Tuning graphene plasmons

The tunable wavelength of graphene plasmons is one of their most significant advantages over conventional metal plasmons [9, 10, 43]. The graphene plasmon wavelength can be tuned by modulating carrier concentrations [10, 44], utilizing coupling effects with substrates [45–48], and deforming graphene [49]. In this section, we will discuss strategies for tuning graphene plasmons.

#### Tuning graphene plasmons via patterning and controlling carrier concentrations

The plasmon resonance wavelength can be tuned by modulating the carrier concentration (i.e., gating, doping) and changing the width of micro-ribbon structures [10]. Plasmon frequency (${\omega }_{p}$) was controlled by micro-ribbon width ($w$) with a relation of ${\omega }_{p}\propto {w}^{-1/2}$, related to characteristics of the 2D electron gas model. For a given micro-ribbon width, the plasmon frequency was controlled using electrostatic gating. The plasmon frequency varied with carrier concentration ($n$), according to the relation ${\omega }_{p}\propto {n}^{1/4}$ [50]. Yan et al. introduced photonic crystal-like structures and demonstrated tunable plasmon frequency in graphene/insulator stacks [44]. In the stacked micro-disks, plasmon resonance frequency was increased with an enhanced intensity in the transmittance spectra due to the strong Coulomb interaction of the graphene layers. However, the plasmon resonant frequency of micro-disks with multiple graphene layers showed a ${n}^{1/2}$dependency, whereas micro-disks with a single graphene layer showed a ${n}^{1/4}$ dependency.

#### Tuning graphene plasmons via coupling with substrate interactions

**Fig. 3:**
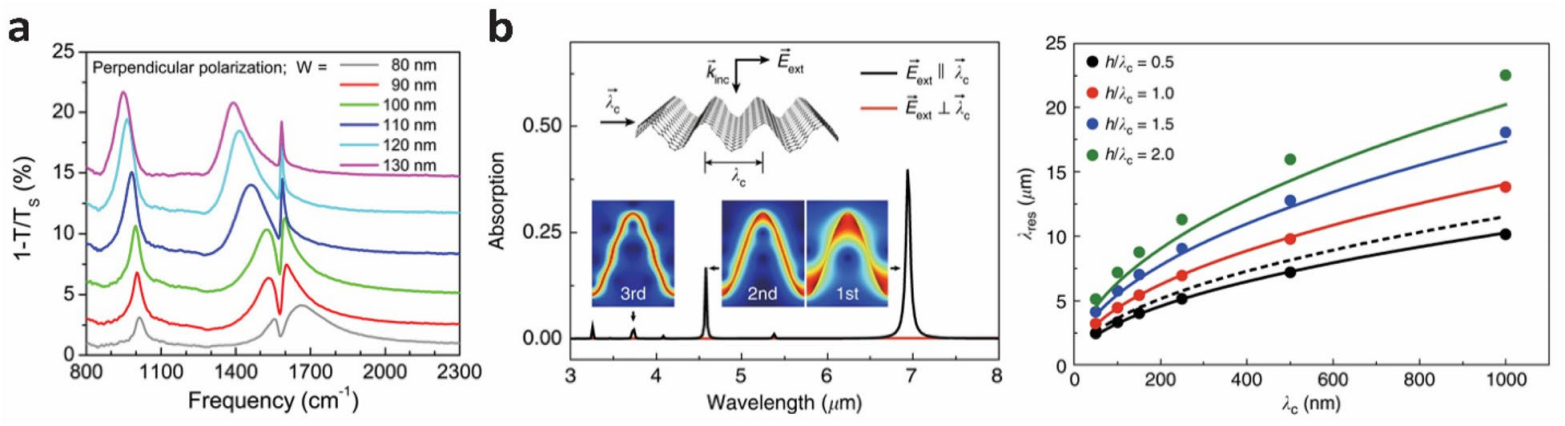
Tuning graphene plasmons by modulating widths of graphene ribbons (**a**) and introducing out-of-plane deformations (**b**). **a** Extinction spectra of bilayer graphene ribbon. The plasmon-phonon coupling is shown by the spectral dip near 1580 cm^− 1^ which depends on the ribbon width. Reprinted with permission from [46]. Copyright 2014 American Chemical Society. **b** Optical absorption spectra of a crumpled graphene structure for a corrugation pitch (${\lambda }_{c}$) of 250 nm and aspect ratio ($h/{\lambda }_{c}$, $h$ refers to the height of the corrugation) of 1 (left). $\overrightarrow{{E}_{ext}}$ denotes the E-field direction of the excitation. Different plasmon resonance modes are shown in the inset. Right panel shows the plasmon resonance wavelength (${\lambda }_{res}$) dependence on the ${\lambda }_{c}$ and aspect ratio. The solid curves represent ${\lambda }_{res}$ calculated from the LC-circuit model and dashed curve represent ${\lambda }_{res}$ estimated from the conventional analytical model. Reprinted under the terms of the Creative Commons License from [49]. Copyright 2018 The Authors. Published by Springer Nature

Graphene plasmons exhibit strong coupling with optical phonons or phonons pertaining to the underlying substrate [45–47]. This coupling effect appears as a dip between the spectral peak in the extinction or transmission spectra known as plasmon/phonon-induced transparency. Such transparency has been reported on AB stacked bilayer graphene nanoribbons where Г-point optical phonons coupled with graphene plasmons [46]. In absence of graphene plasmon excitation, a symmetric phonon absorption peak was observed at 1580 cm^− 1^. However, the spectral peak became asymmetric with a much higher extinction intensity when it was coupled with the graphene plasmon peak. The transparency in the spectra between the broad plasmon peak and sharp phonon peak was prominent. The transparency window (i.e., the gap between the spectral peaks) became narrower as the graphene plasmon frequency was tuned by the nanoribbon width causing the frequency to approach the phonon frequency of AB stacked bilayer graphene (the spectral dip near 1580 cm^− 1^ in Fig. 3a). The transparency window was tuned simply by changing the nanoribbon width. A similar observation was reported on a graphene-hBN nanoribbon structure where graphene plasmons coupled with the hBN phonons [47].

The coupling between graphene plasmons and phonons from the underlying material often exhibits surface plasmon-phonon polaritons (SPPPs) [45, 48]. Brar et al. reported SPPP mode induced by plasmon-phonon hybridization in graphene-hBN heterostructures [45]. The hybridization was explained using an electromagnetically coupled oscillator model. In their graphene-hBN heterostructure, the hBN underwent lattice displacement and created local polarization fields, which induced near-field interactions with free carriers in the graphene layer. Similarly, the local polarization fields in the graphene exerted forces on the hBN lattice. Dai et al. investigated the plasmon-phonon hybridization in a graphene-hBN heterostructure by direct nano-IR imaging and demonstrated tunability of the hyperbolic phonon-polaritons in the hBN layer using SPPP [48]. Within the heterostructure, SPPs in graphene and hyperbolic phonon polaritons in hBN were hybridized and revealed a new collective mode: hyperbolic plasmon-phonon polaritons. The hyperbolic plasmon-phonon polaritons exhibited hyperbolic response (h-BN phonon-like response) in addition to gate tunability (graphene plasmon-like response).

#### Tuning graphene plasmons via structural deformation

Graphene plasmon resonance can be tuned by introducing three-dimensionality to graphene [49]. Kang et al. reported that the plasmon resonance in a crumpled graphene was highly dependent on the direction of the incident E-field. Specifically, when the E-field was perpendicular to the crumpling direction vector ($\overrightarrow{{\lambda }_{c}}$, defined in Fig. 3b), a finite element model predicted no plasmon resonance. On the other hand, when the E-field was parallel to the crumpling direction vector, the model predicted multiple plasmon resonance peaks (left panel of Fig. 3b). When the crumpling wavelength (${\lambda }_{c}$, defined in Fig. 3b) was fixed, the plasmon wavelength, optical absorption, and extinction all increased with increasing corrugation aspect ratio (aspect ratio defined as height ($h$)/wavelength (${\lambda }_{c}$) of the corrugation) (right panel of Fig. 3b). On the other hand, when the aspect ratio was fixed, the plasmon wavelength, optical absorption, and extinction all increased with increasing crumpling wavelength. This graphene plasmon behavior was explained using an analogous LC circuit model, where L and C represent the inductance and capacitance of a non-parallel plate capacitor. One period of graphene corrugation was modeled as a non-parallel plate capacitor, and the periodic corrugation was modelled as capacitors connected in series. In the model, the inductance and capacitance represented the current and charge induced by the graphene plasmons, respectively. The model suggested that the inductance was a function of ${\lambda }_{c}$ while the capacitance was a function of the aspect ratio of the corrugation. Although the plasmon wavelength blueshifted with the increase in Fermi energy of the graphene, the plasmon wavelength was more sensitive to geometrical characteristics of the corrugation than change in Fermi energy. Thus, this work suggested that modulating the geometries of graphene can be a more effective method for tuning graphene plasmons when compared to controlling doping levels.

## Plasmonic effect of graphene-based hybrid materials

While graphene plasmons exhibited extraordinary intrinsic properties originating from high electron mobility, low loss of plasmons, high spatial confinement, and wide range of tunability, graphene has been combined with conventional plasmonic nanostructures for the enhanced sensitivities, sensing in visible wavelength, and more. In graphene-based hybrid plasmonic materials, graphene plays various roles owing to its smooth surface without dangling bonds, atomic thickness, chemical inertness, gas impermeability, and optical transparency. Moreover, graphene is a fluorescence (FL) quencher while contributing to surface enhanced Raman scattering (SERS) effects, which makes graphene an attractive option for sensing platforms [51–54]. In this section, we discuss graphene-based hybrid materials with a specific focus on roles of graphene in hybrid materials and strategies for creating hybrid materials.

### Graphene as a fluorescence quencher and Raman enhancer

One challenge in characterizing a target molecule using Raman spectroscopy is suppressing the target molecule’s FL emission when the target molecule exhibits high FL emission at a particular laser excitation. The high FL emission masks the Raman signals of the target molecule, making those signals difficult to detect. For example, rhodamine 6G (R6G) emits strong FL signals at 633 nm excitation wavelength, and the intensity of the FL signal is much larger than its Raman signal. However, graphene has shown its potential not only as an effective surface for Raman enhancement but also as a FL quencher.

As discussed, graphene is virtually transparent, absorbing only ~ 2% of incident light regardless of its wavelength in the visible range [55], and acts as a semimetal, exhibiting extremely high carrier mobility [56]. Because graphene’s optical absorbance is nearly constant across the visible range, and it shows a linear band dispersion at the corners of the Brillouin zone, excitation of electron-hole pairs in graphene allows quenching of optically or electronically excited species by resonant energy transfer [51, 52]. Xie et al. reported that FL emitted by R6G and protoporphyrin IX (PPP) molecules were quenched by exfoliated monolayer graphene [53]. At 514 and 633 nm laser excitation wavelengths, the Raman peaks of R6G and PPP molecules, respectively, could not be observed due to the high FL signals and FL noise level when the molecules were in solution. However, the FL signals were reduced by a factor of ~ 10^3^ when the R6G and PPP molecules were adsorbed on graphene, allowing the Raman peaks to be observed. Similar effects have been reported elsewhere [51, 52]. Graphene not only suppresses FL signals but also enhances Raman signals of target molecules [53, 54].

**Fig. 4:**
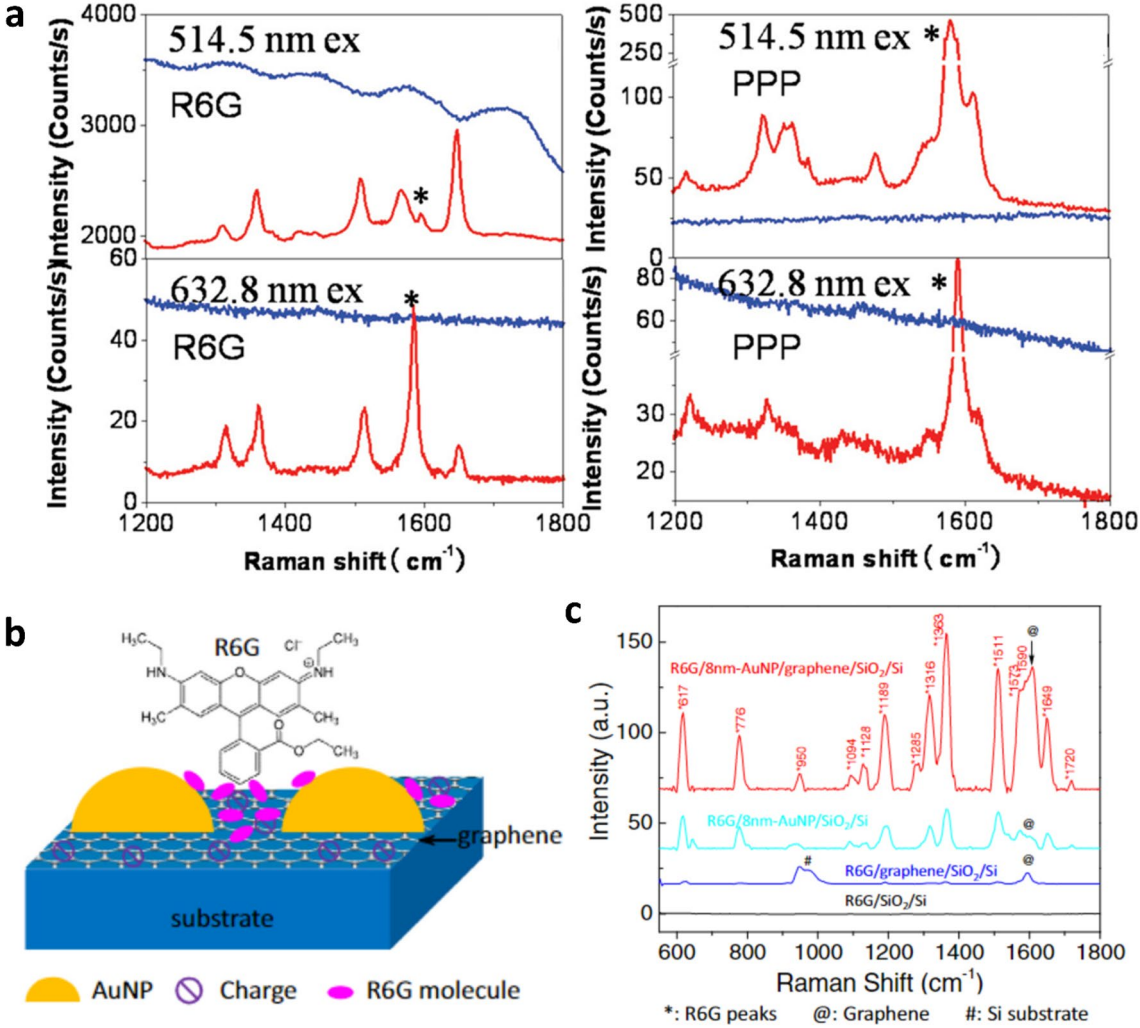
Roles of graphene in graphene-based plasmonic materials. **a** Graphene as a FL quencher: comparisons of Raman signals of R6G and PPP deposited on graphene (red line) and on the SiO_2_/Si substrate (blue line) at 514.5 nm excitation and 632.8 nm excitation. The asterisk (*) indicates G-band peak of graphene. Reprinted with permission from [54]. Copyright 2010 American Chemical Society. **b**, **c** Graphene as a substrate material for hybrid structures: **b** Schematic diagram of R6G molecules attached on AuNPs/graphene/SiO_2_/Si substrate for SERS, **c** Raman spectra for R6G on AuNPs/graphene/SiO_2_/Si (red), AuNPs/SiO_2_/Si (cyan), graphene/SiO_2_/Si (blue), and bare SiO_2_/Si (black). Reprinted from High sensitivity surface enhanced Raman spectroscopy of R6G on in situ fabricated Au nanoparticle/graphene plasmonic substrates, Rongtao Lu et al., Copyright 2015, with permission from Elsevier [64]

Ling et al. proposed the possibility for graphene as a substrate for Raman enhancement based on chemical mechanisms (CM) [54]. In order to trigger CMs for Raman enhancement, the distance between graphene and the target molecules should be very close (less than 0.2 nm), and the Fermi level of the graphene should be in between the highest occupied molecular orbital (HOMO) and the lowest unoccupied molecular orbital (LUMO) of the target molecules to boost charge transfer. Phthalocyanine (Pc), R6G, PPP and crystal violet (CV) satisfy this orbital energy alignment and showed much stronger Raman signals on exfoliated graphene than on SiO_2_/Si substrates. Specifically, as shown in Fig. 4a, it was impossible to observe the Raman peaks from both R6G and PPP on SiO_2_/Si substrate at 514.5 and 632.8 nm excitations. However, placing a graphene layer underneath these molecules enabled clear observation of the Raman peaks of both R6G and PPP molecules by quenching the FL emission and enhancing the Raman signals of the molecules. The FL quenching and Raman enhancing capabilities of graphene with its optical, electromagnetic, and chemical characteristics allow graphene to be utilized as a component of various plasmonic hybrid materials.

### Roles of graphene in plasmonic hybrid materials

Conventional metallic nanomaterials such as metal nanowires, nanodisks, NPs, and nanopyramids on dielectric substrate have been introduced for various plasmonic applications due to their strong local electromagnetic field enhancement near the rough metal surface [57, 58]. Although graphene is a promising plasmonic material, its flat surface inhibits strong electromagnetic field enhancement. Therefore, integrating metallic nanomaterials with graphene to form hybrid structures offers the enhanced plasmonic properties of graphene while maintaining graphene’s distinctive features such as its uniform subnanometer-scale thickness, robust chemical resistance, and FL quenching effect. This section discusses different roles of graphene in metal nanostructure-graphene hybrid plasmonic materials. In hybrid materials, graphene serves as: (i) a substrate layer for the overlaying structure, (ii) a protective layer for the underlying structure, and (iii) a gap material between the two structures. Graphene as a substrate provides a smooth surface, quenches FL signals, and improves Raman signals of target molecules. Graphene as a protective layer enhances chemical and thermal stability of underlying materials. Lastly, graphene as a gap material offers angstrom-scale precise gap control for metal-graphene-metal hybrid structures.

As previously discussed in Sect. 3.1, graphene can itself be utilized as a substrate for Raman enhancement [54, 59]. In addition, a graphene layer can provide high affinity to aromatic- and bio-molecules [59, 60]. However, conventional plasmonic nanomaterials, e.g., noble metal nanostructures, are often more advantageous for Raman enhancement. Noble metal nanostructures excite surface plasmons by absorbing visible light, while a rough nanostructure surface induces highly localized electromagnetic fields resulting in larger Raman enhancement factor via electromagnetic mechanism (EM), especially for sensing applications. Raman signals can be amplified by a factor of 10^8^ [54, 60–62]. In contrast, graphene has a relatively smooth surface and limited absorption capability for visible light. In order to combine graphene’s FL quenching and CM enhancement with metal’s EM enhancement, metal-graphene hybrid structures have been proposed and investigated. Au [63–72], Ag [65, 73, 74], and Cu [75, 76] are the most frequently used metals. As an example, Lu et al. demonstrated in situ fabrication of AuNPs on chemical vapor deposition (CVD)-grown graphene for SERS studies as illustrated in Fig. 4b [64]. The size of the AuNPs was controlled by nominal Au thickness. As illustrated in Fig. 4c, no peak was observed from R6G molecules on a bare SiO_2_/Si wafer from the Raman spectrum in the range of 600–1800 cm^− 1^. However, on a graphene/SiO_2_/Si substrate, Raman peaks from R6G were slightly visible due to graphene’s CM effects, and significantly increased Raman peaks were observed with AuNPs/ SiO_2_/Si and AuNPs/graphene/SiO_2_/Si substrates. Based on the size of the peak located at 1363 cm^− 1^, which is the feature peak of R6G, R6G molecules on AuNPs/SiO_2_/Si and AuNPs/graphene/SiO_2_/Si exhibited between 21 and 86 times greater peak intensity, respectively, compared to R6G molecules on graphene/SiO_2_/Si substrate. These results suggest that the graphene layer provided additional enhancement via the CM effect while suppressing FL signals.

In addition to flat substrates such as wafer or glass, metal-graphene hybrid SERS substrates have been developed with polymers for three-dimensional (3D) structuring [69, 72, 77] and flexibility [78–80]. Leem et al. reported mechanically self-assembled AuNPs on 3D structured graphene/polystyrene (PS) substrates [72]. The 3D structured AuNPs/graphene hybrid substrates showed at least 10 times higher Raman enhancement when compared to flat AuNPs/graphene substrates due to denser nanoplasmonic “hot spots” and reduced distances between AuNPs by 3D structuring. Xu et al. first demonstrated a poly(methyl methacrylate) (PMMA)/AuNPs/graphene hybrid structure as a graphene-SERS tape floating on solution [79], and Dong et al. further developed an AuNPs/graphene/AuNPs sandwich structure with polyethylene (PE) film as a flexible SERS substrate [80].

Graphene also can be used as a substrate for tunable plasmonic hybrid materials. Niu et al. controlled the SPR of AuNPs/Al_2_O_3_/graphene hybrid structures by changing the thickness of the Al_2_O_3_ layer [81]. Without a graphene layer, the resonance wavelength of localized surface plasmons was maintained at 569 nm in transmittance spectra irrespective of Al_2_O_3_ layer thickness. On the other hand, the resonance wavelength of the AuNPs/Al_2_O_3_/graphene shifted from 583 to 566 nm as the thickness of the Al_2_O_3_ layer varied from 0.3 to 1.8 nm, implying the formation of oscillating image dipoles within the graphene substrate.

#### Graphene as a protecting layer of metal nanostructures

Metal NPs often suffer from the poor stability due to photoinduced damage by laser illumination and oxidation in air [82, 83]. However, graphene is chemically stable and impermeable even to helium gas molecules, and graphene films can therefore be used to cover and protect vulnerable nanostructures made from metal NPs [84, 85].

**Fig. 5:**
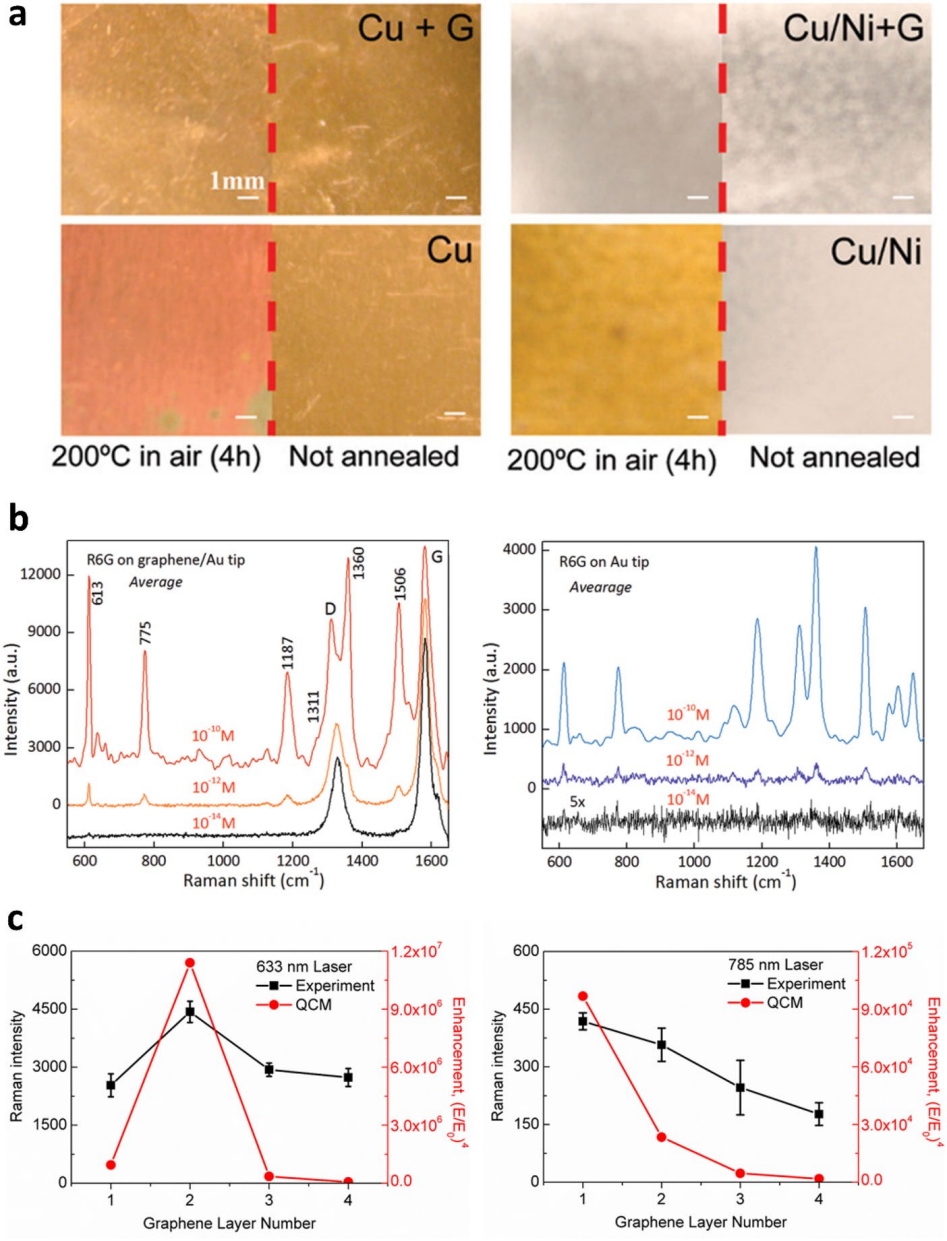
Roles of graphene in graphene-based plasmonic materials. **a** Optical images of Cu and Cu/Ni foil with and without graphene coating taken before and after annealing in air (200 °C, 4 h). Reprinted with permission from [84]. Copyright 2011 American Chemical Society. **b** Raman spectra of R6G on graphene hybrid structure with 3 different concentrations (10^− 10^ M, 10^− 12^ M, and 10^− 14^ M) and Raman spectra of R6G on Au tips with 3 different concentrations. Reproduced with permission from [71] Copyright 2013 WILEY-VCH Verlag GmbH & Co. KGaA, Weinheim. **c** Graphene as a gap material enabling the thickness control with the sub-nanometer scale precision: comparison between the experimental (black) and computed (red) optical responses for samples excited by a 633 nm laser and a 785 nm laser. The left vertical axis represents the peak Raman intensity measured from a solution of brilliant cresyl blue molecules. The different layer number of graphene gap significantly changes the Raman enhancement of the whole hybrid system. Reprinted under the terms of the Creative Commons License from [94]. Copyright 2019 The Authors. Published by Springer Nature

Chen et al. demonstrated oxidation resistance of graphene-coated metal films [84]. Monolayer and few-layer graphene sheets were grown by CVD on Cu and Cu/Ni alloy foils, respectively. After heating at 200 °C in air for 4 h, the graphene coated metal foils remained unoxidized, while uncoated Cu and Cu/Ni foils were oxidized. The enhanced oxidation resistance was easily observable with the naked eye as shown in Fig. 5a. Following this work, a number of studies were carried out, reporting the effectiveness of graphene coatings for preventing oxidation of Au, Ag, and Cu NPs [60, 74–76, 82, 83, 86, 87].

Liu et al. encapsulated Cu, Ag, and Au NPs under few-layer graphene to improve oxidation and photobleaching resistances of metal NPs for SERS applications [82]. Cu, Ag, and Au thin metal films were deposited by thermal evaporation and annealed to form metal NPs. Next, few-layer graphene was grown on the surface of the metal NPs by CVD. The stabilities of the graphene covered metal NPs were demonstrated with ultraviolet–visible (UV-vis) spectroscopy and Raman spectroscopy. First, Ag and Cu NPs covered by graphene maintained their UV-vis spectra up to 20 h (Ag) and 1 h (Cu) of air exposure durations, whereas UV-vis spectra of bare Ag and Cu NPs exhibited notable redshifts of the SPR peak within 1 h (Ag) and 10 min (Cu) of air exposure. This implies that the graphene coating significantly slowed the oxidation of metal NPs. Additionally, graphene layers were found to enhance Raman sensing by reducing the photobleaching effect. Raman signals of cobalt phthalocyanine (CoPc) on bare AuNP substrates slowly degraded under 160 s of laser excitation, while graphene covered AuNP substrates maintained the CoPc Raman signals under the same excitation conditions. Moreover, graphene covered AuNP substrates exhibited boosted CoPc Raman signal intensity, demonstrating the Raman enhancement capabilities of graphene as a passivation film.

In addition, graphene has also been demonstrated as a protective layer for metal NPs from thermal degradation. Zhang et al. developed Au triangular nanoarrays (TNAs) covered by monolayer graphene [87]. Under annealing temperatures up to 400 °C, the graphene layer effectively protected Au TNAs from thermal degradation. Furthermore, the graphene-coated Au TNAs hybrid substrates were reusable for Raman enhancement up to 15 times via detection and cleaning (30 min-long, 300 °C annealing under Ar atmosphere) cycles. Graphene coatings were additionally applied to enhance chemical stability under corrosive environments by passivating Ag nanostructures [60].

In addition to the improved stability and repeatability, an increase in Raman intensity for SERS applications is expected for graphene-protected NP hybrid structures. Wang et al. developed a monolayer graphene covered Au nanopyramid hybrid system [71]. The hybrid system exhibited excellent detection capabilities, as low as 10^− 14^ M and 10^− 12^ M concentrations for R6G and lysozyme molecules, respectively. Moreover, the hybrid system showed approximately 10 times greater SERS intensity when compared to bare Au nanopyramids, demonstrating Raman enhancement induced by the graphene coating layer (Fig. 5b). Liu et al. developed monolayer graphene/Ag-coated silica nanosphere arrays as SERS substrates [60]. In comparison to the uncoated SERS substrates (Ag-coated silica nanosphere arrays), the graphene-coated SERS substrates demonstrated up to a twofold increase in Raman intensity for CV and R6G.

#### Graphene as a gap material for plasmonic hybrid structures

Under light illumination, two adjacent metal nanostructures can generate highly concentrated electromagnetic fields inducing tremendous Raman enhancement [88, 89]. The subnanometer plasmonic gap between two different metal structures plays an important role in plasmonic applications [90–92]. However, it has been a significant challenge to control the plasmonic gap at the angstrom scale. The thickness of the monolayer graphene is ~ 0.34 nm, which provides precise thickness control at the sub-nanometer scale. Compared to metals, graphene is a relatively less conductive layer due to its low through-plane conductivity [93, 94].

Mertens et al. demonstrated a monolayer graphene spacer between a 100 nm Au film and a 80 nm AuNPs [92]. With minimal gap (AuNPs on Au film), the resonance frequency of the charge-transfer plasmons was 720 nm. Upon adding a graphene spacer however, the resonance frequency shifted down to 670 nm with 1- to 7-layer graphene gaps providing precise tunability of the plasmon resonances. Li et al. further observed Raman enhancement with an AgNPs/graphene/Ag film hybrid structure [95]. Based on a conventional understanding of SERS enhancement via EM mechanism, plasmonic enhancement always increases as gap thickness decreases. However, Lee et al. investigated the quantum plasmon tunneling effect on AuNPs/graphene/Au films and suggested that the plasmonic field enhancement by a sub-nanometer gap can be significantly reduced when the gap is short enough to trigger quantum tunneling [94]. As illustrated in Fig. 5c, the experimental and computational results showed that AuNPs/graphene/Au film hybrid structures exhibit increasing Raman intensity as the graphene gap becomes thinner, which is consistent with the conventional trend, while the same hybrid structure exhibits a sudden drop in Raman intensity with a monolayer graphene spacer at 633 nm excitation.

In addition to metal NPs/graphene/metal film hybrid structures, AgNPs/graphene/AgNPs [96] and AuNPs/graphene/AuNPs [97] hybrid structures were also demonstrated. Both hybrid structures exhibited highly enhanced E-field distribution between the metal NPs and showed significantly enhanced Raman signals at metal NP/graphene/metal NP locations with an enhancement factor of 20–300 when compared to the graphene substrate alone. Zaretski et al. further utilized graphene as a template for subnanometer dielectric gaps and fabricated Au/air gap/Au structures by post-etching the graphene layer [98]. Compared to single Au wires, the Au wire-air gap-Au wire showed 50 times greater Raman signal from benzenethiolate. A rippled AuNPs/graphene/AuNPs structure has been developed by Lee et al. to allow additional plasmonic enhancement by reducing the distance between AuNPs and densifying the “hot-spots” [77].

### Tunability of graphene-based hybrid materials

The plasmon resonance of graphene-based hybrid materials can be controlled both electrically and structurally. Electrical tuning is primarily based on changing a gate voltage [99–102], whereas structural tuning can be performed by varying the size of metal nanostructures [64, 102, 103], the distance between metal nanostructures [78], and the thicknesses of substrate, gap, and protective layers [81, 92, 101, 104].

**Fig. 6:**
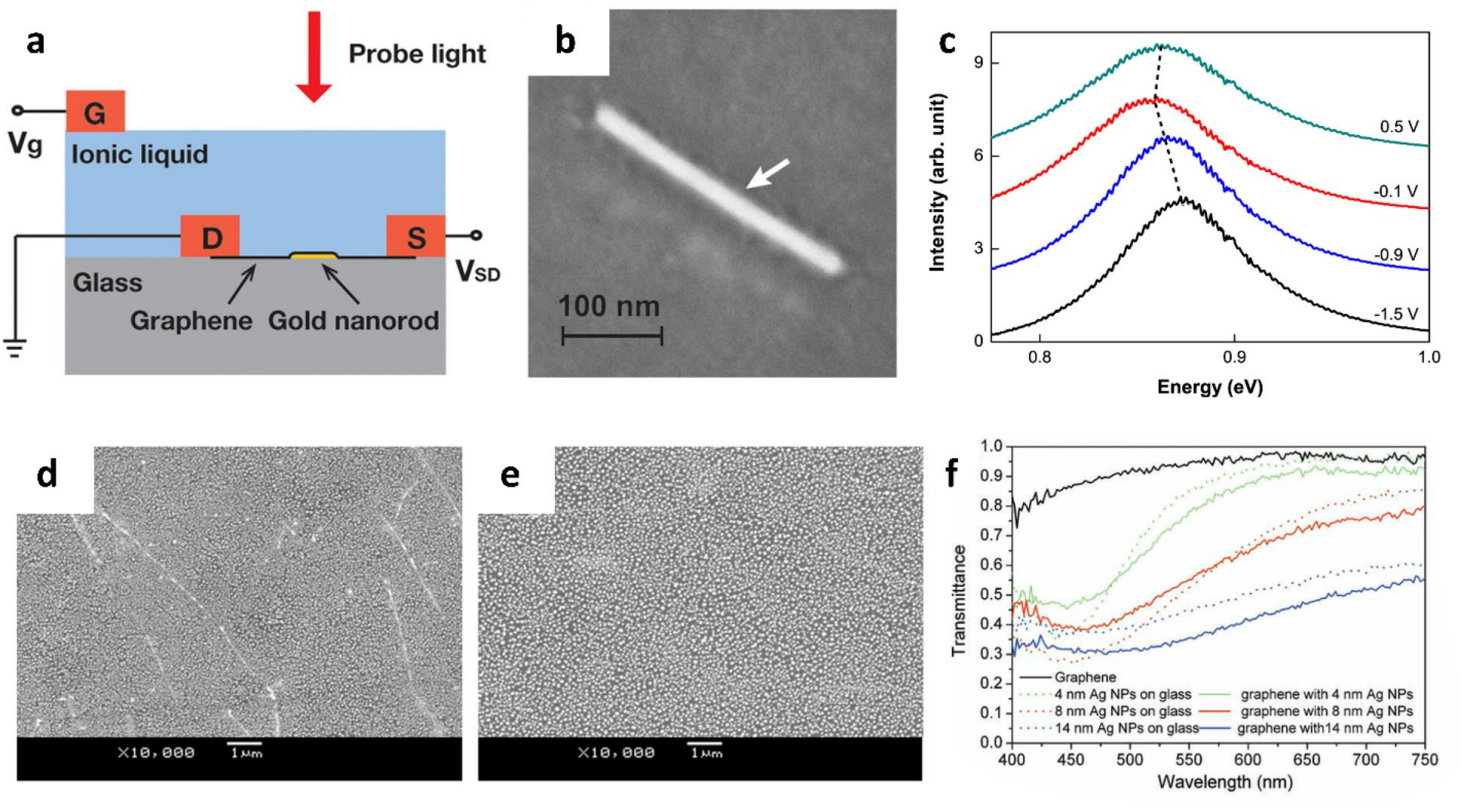
Tunability of graphene-based plasmonic materials. **a–c** Electrical tunability of graphene/Au nanorod hybrid structure. **a** Schematic diagram of a graphene/Au nanorod hybrid structure gated through ionic liquid. A top electrolyte gate was used to control plasmon resonance of the hybrid system by varying optical transitions in graphene. **b** A high-resolution scanning electron micrograph (SEM) image of the graphene/single Au nanorod. Graphene draped over the nanorods (white arrow). **c** Rayleigh scattering spectra of the graphene/Au nanorod structure at 0.5, − 0.1, − 0.9, and − 1.5 V of gate voltage. The plasmon resonance shifted depending on the gate voltage. Reprinted with permission from [99]. Copyright 2012 American Chemical Society. **d**, **e** Structural tunability of AgNPs/graphene hybrid structure. AgNPs with **d** 49.3 nm and **e** 67.5 nm of lateral dimension on graphene formed through thermal annealing of 4 and 8 nm thick Ag films, respectively. **f** Transmittance spectra taken on graphene (black) and AgNPs/graphene hybrid structures (colored solid lines). SPR of AgNPs/graphene structures redshifts with increasing lateral size of the AgNPs. The 4, 8, and 14 nm AgNPs in label indicate the thickness of as-deposited Ag film which were transformed to AgNPs with 49.3, 67.5, and 147.5 nm of lateral size, respectively, after dewetting. Reproduced with permission from [103]. Copyright 2012 WILEY-VCH Verlag GmbH & Co. KGaA, Weinheim

Kim et al. reported plasmon resonance tunability of graphene/Au nanorod hybrid structures via gate voltage [99]. An Au nanorod on glass substrate was encapsulated with CVD grown graphene, and electrostatic gating was applied on the graphene through an ionic liquid, 1-ethyl-3-methylimidazolium bis(trifluoromethylsulfonyl)imide (Fig. 6a, b). From dark-field Rayleigh scattering spectroscopy, the plasmon resonance peak varied from 0.863 eV (1437 nm) to 0.857 eV (1447 nm), and to 0.875 eV (1417 nm) as the gate voltage changed from 0.5 V to − 0.1 V, and to − 1.5 V (Fig. 6c). The decreasing and increasing resonance energy was attributed to a gate-dependent complex dielectric constant of the graphene which consists of real and imaginary parts. The real part becomes maximum when the gate-shifted graphene Fermi energy, E_F_, meets the condition of 2|E_F_| = 0.86 eV, where 0.86 eV is the plasmon resonance of a bare Au nanorod. On the other hand, the imaginary part shows a stepwise decrease at 2|E_F_| = 0.86 eV. As a result, the plasmon resonance of the graphene/Au nanorod hybrid structures exhibited lowest resonance energy at 2|E_F_| = 0.86 eV, which occurred under − 0.1 V of gate voltage.

In order to structurally tune the plasmon resonances of the graphene-based hybrid materials, Xu et al. developed different sizes of AgNPs on graphene and measured the optical transmittance [103]. As shown in Fig. 6f, The SPR wavelength redshifted from 446 to 495 nm as the average lateral dimension of the AuNPs increased from 49.3 to 147.5 nm. Similar results were reported with AuNPs on graphene, and the plasmon resonance wavelength also redshifted from 606 to 654 nm with increasing mean dimension of AuNPs from 13.6 to 46.2 nm [64]. For both studies, size control of nanostructures was achieved simply by changing the thickness of the as-deposited metal film before dewetting. Another simple way to tune plasmon resonance is to modify the thicknesses of the substrate, gap, or protective layers. Xu et al. also varied the layer number of the graphene substrate from 1- to 3-layers for AgNPs and observed a plasmon resonance shift from 424 to 440 nm [103]. This redshifting trend with increasing graphene layer number was also observed from graphene coated AuNPs [104]. However, the trend of resonance shifting by modifying the gap layer thickness was not always unidirectional, due to the quantum tunneling effect mentioned previously in Sect. 3.2. The plasmon resonance of metal/gap layer/metal hybrid structures usually redshifts as the thickness of the gap narrows [81, 91], but the resonance blueshifts or disappears when the gap becomes thin enough for quantum tunneling [92, 94].

## Plasmonic sensing applications

Compared to conventional plasmonic materials such as noble metals, graphene plasmons exhibit ultra-high electromagnetic field confinements with longer lifetimes. These extraordinary plasmonic properties of graphene are sensitive to changes in the surrounding dielectric environments. In addition, graphene’s plasmonic wavelengths are tunable by applying gate voltages or via chemical doping, which makes it attractive for sensing applications [7–9]. In this section, we will discuss graphene-based plasmonic sensing applications in various fields including biosensors, chemical sensors, optical sensors, and other types of sensors. For each topic, we will introduce recent works based on graphene plasmonic structures, as well as hybrid structure of graphene with other plasmonic materials.

### Biosensors

**Fig. 7:**
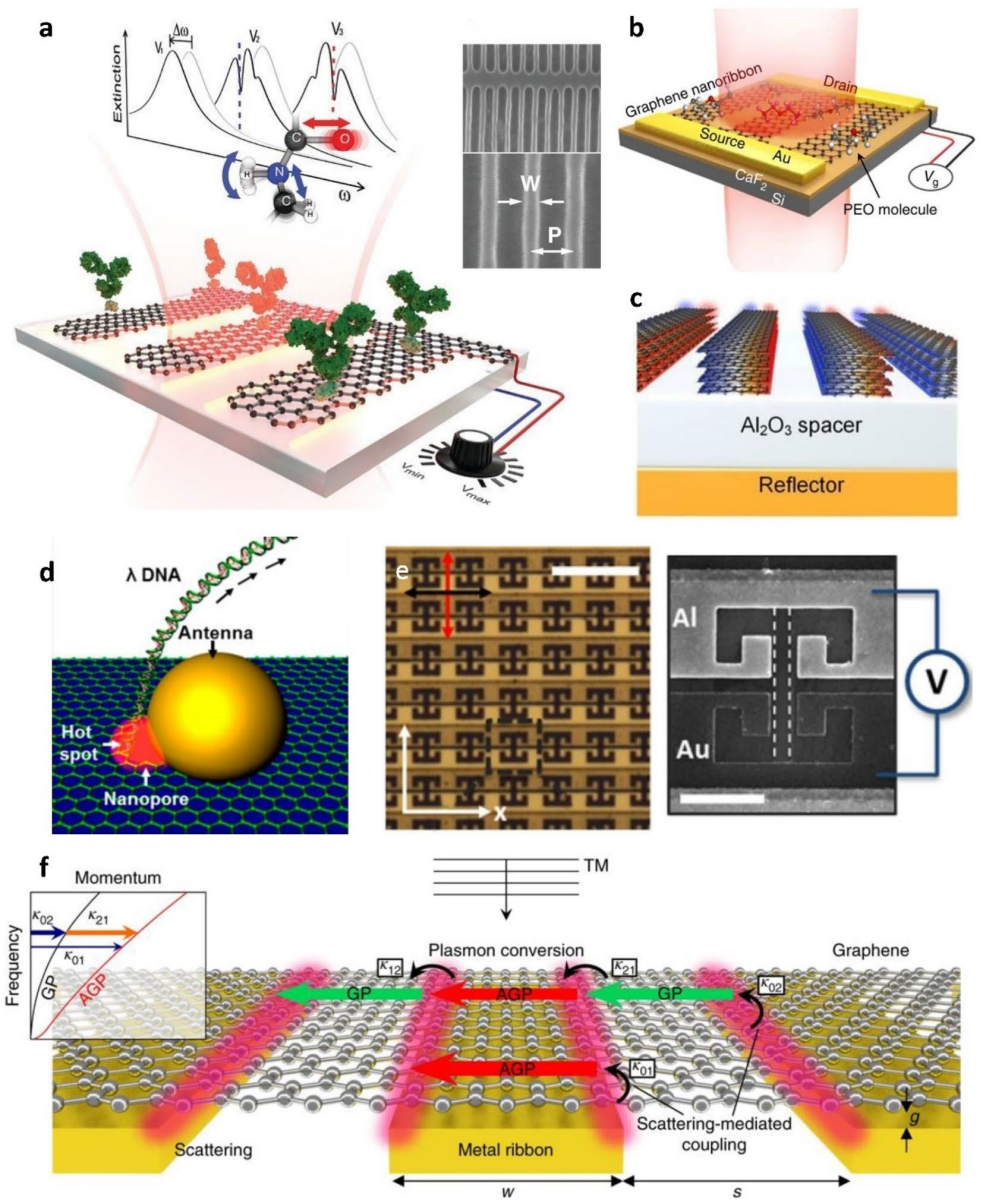
Graphene-based plasmonic sensors for biosensing. **a** Graphene plasmonic biosensor for protein. The proteins adsorbed on the graphene surface were detected by resonance frequency shift accompanied by dips of protein vibration bands. From [7]. Reproduced with permission from AAAS. **b** Schematic illustration of graphene nanoribbon sensor on a CaF_2_ dielectric substrate. CaF_2_ dielectric layer was introduced to reduce plasmon-phonon hybridization between graphene and the substrate. Reprinted under the terms of the Creative Commons License from [12]. Copyright 2016 The Authors. Published by Springer Nature. **c** Plasmon enhanced by FP-like cavity and multilayer graphene. Reproduced with permission from [106]. Copyright 2020 Wiley-VCH GmbH. **d** Plasmonically enhanced FL for DNA sensing while DNA is passing through a graphene nanopore integrated with an Au nanoantenna. Reproduced with permission from [108]. Copyright 2014 American Chemical Society. **e** Optical (left) and SEM (right) images of graphene nanoribbon sensors in the gap of CSRR. Scale bars are 10 μm and 2 μm in left and right figures respectively. Reproduced under the terms of the ACS AuthorChoice License from [116]. Copyright 2016 American Chemical Society. **f** Schematic illustration of the acoustic plasmon resonator architecture and coupling routes to plasmon modes for a planewave normally incident with TM polarization. Red and green arrows represent acoustic graphene plasmons (AGPs) and conventional graphene plasmons (GPs), respectively. Reprinted by permission from Springer Nature Customer Service Centre GmbH: Springer Nature, Nature Nanotechnology, Graphene acoustic plasmon resonator for ultrasensitive infrared spectroscopy, Lee et al., Copyright 2019 [107]

Localized plasmons in graphene nanostructures have resonance frequencies in the mid-IR range, overlapping with the vibrational frequencies of many biological and organic molecules. Additionally, graphene is biocompatible and chemically inert under most physiological conditions. Graphene-based plasmonic nanostructures therefore offer substantial potential for sensing biomolecules, viruses, micro-organisms, and cells. Li et al. were among the first to experimentally demonstrate plasmonically enhanced light-matter interactions of graphene-based nanostructures at the mid-IR range [11]. Their sensors were made from graphene nanoribbons with PMMA or poly(vinylpyrrolidone) (PVP) deposited via solution processes on top of the graphene as analytes. These polymers have fingerprints in the mid-IR region where most biomolecules likewise exhibit their fingerprint spectra. When the graphene nanoribbons were illuminated with IR light, highly confined electromagnetic fields near the edges of the graphene nanoribbons enhanced the interaction between the IR light and the molecular adsorbates on the graphene surface. As a result, the optical absorption was enhanced by a factor of 5. Rodrigo et al. used graphene SPRs in graphene nanoribbon arrays to detect proteins (Fig. 7a) [7]. When proteins were adsorbed on the graphene nanoribbons, the resonance frequency of graphene plasmons shifted by more than 200 cm^− 1^ depending on the gate voltage. In comparison to Au nanorod antenna arrays, the graphene plasmonic sensors showed 6 times higher resonance frequency shift and 3 times greater sensitivity towards proteins. In addition, Cai et al. investigated the effects of graphene ribbon widths and gate potentials applied to graphene ribbon biosensors. Enhanced sensitivities towards proteins and lipids were demonstrated through numerical simulations [105].

The resonance frequencies of graphene plasmons are thought to be restricted by substrate effects due to plasmon-phonon hybridization, which restrains the propagation of plasmon energy at graphene-substrate interfaces. Hu et al. placed CaF_2_ dielectric nanofilms between graphene nanoribbons and their silicon substrates (Fig. 7b) [12] to avoid plasmon-phonon hybridization between the graphene and silicon substrate and enable electrically tunable plasmonic effects encompassing the full molecular fingerprint region. Polyethylene oxide (PEO) was employed as an analyte because it has been widely studied and possesses a wide fingerprint spectrum in the mid-IR and far-IR regimes, overlapping with the target fingerprints for most food sensing and biosensing applications. This configuration enabled PEO detection with 20 times enhanced sensitivity.

While graphene is expected to exhibit high plasmonic enhancement factors in theory, experimental demonstration has been limited due to the low IR absorption of graphene. Recently, Nong et al. reported that the absorptions of light and mode energy (total local field energy at graphene plasmon resonance) of graphene plasmons were improved by employing Al_2_O_3_ Fabry-Perot (FP) like cavities and multilayer graphene nanoribbons (Fig. 7c) [106]. Consequently, the absorption of their sensors increased from 3 to 92%, and an enhancement factor of 162 was achieved.

Furthermore, hybrid structures of graphene with other plasmonic materials have been widely investigated for optimally utilizing not only the materials’ individual advantageous properties but also their synergistic functionalities. Specifically, combining plasmonic materials with graphene can compensate for the mismatch between the wave vectors of graphene plasmons and incident light, thereby expanding the detectable wavelength range. In addition, graphene can enhance SPR signals due to strong electromagnetic field confinement in hybrid materials [107].

In hybrid structures, nanostructures of noble metals (e.g., Au, Ag) are commonly combined with graphene. Graphene can serve as either a substrate or a protective layer for vulnerable materials, while metal nanostructures serve as nanoantennas [77]. Nam et al. reported that graphene nanopores integrated with AuNPs could be used for detecting DNA translocation events (Fig. 7d) [108]. The nanopores and Au NPs were simultaneously formed via the photothermal effect when illuminating laser on Au nanorods on graphene with a femto-second laser. The plasmonic resonance of the AuNPs overlapped with the absorption peak of the FL signal from the fluorescently labelled DNA and thereby enhanced the optical signal during DNA translocation by 4.5 times. Lee et al. decorated graphene flakes with reduced AuNPs and modified the AuNP surfaces with antibodies [109]. The resulting heterostructures allowed them to detect a tuberculosis antigen, CFP-10, with adetection limit of 4.5 pg/mL. AgNPs have also been widely used in graphene-based hybrid plasmonic materials for sensing biomolecules, such as phosphate buffered saline (PBS), DNA, dopamine (DA), and glutathione (GSH) [110, 111].

Alternative materials have been proposed as replacements for noble metals in these hybrid structures, as noble metals were found to sometimes induce defects in the graphene lattice due to chemical interactions [112, 113]. Pau et al. reported a strong plasmonic effect with GaNPs-graphene hybrid materials. Compared to other noble metals, Ga exhibits weaker bonding with graphene, is less destructive to the graphene lattice, and is more stable in ambient environments. GaNPs on continuous graphene were used to detect the bio-functionalizing agent 3,30-dithiodipropionic acid di(N-succinimidyl ester) (DTSP), and an obvious resonance shift was observed after chemical modification [114]. Liu et al. proposed a different kind of dielectric NP, PS particles, to decorate graphene surface [115]. These dielectric particles were found to enhance the absorption of mid-IR range light and confine electromagnetic fields in the same manner as metal NPs. Their resulting hybrid graphene-dielectric particle sensors were able to successfully recognize the vibrational modes of biomolecule para-aminobenzoic acid (PABA).

Graphene has also been integrated with other types of plasmonic structures, such as optical metamaterials or metal gratings, for biosensing applications. Luxmoore et al. demonstrated a graphene nanoribbon sensor placed inside the gap of a complementary split ring resonator (CSRR) (Fig. 7e) [116]. CSRRs confine E-fields inside gaps thereby enhancing the electromagnetic absorption of graphene nanoribbons. Additionally, the dissimilar metal composition of the resonator leads to an asymmetry carrier density and Seebeck coefficient along the graphene channel. This produces a one to two order of magnitude enhancement in response and enables direct electrical read-out by measuring the photovoltages between the ends of the graphene ribbon. This hybrid metamaterial detector was applied for detecting PMMA. Additionally, other types of metamaterial designs, including Au circles and split ring resonators, were employed as graphene decorations for hemoglobin and urine biomolecule detection [117]. Patterned metal gratings were another type of structure integrated with graphene to enhance the plasmonic absorption for biomolecular detection. Wu et al. used Au grating substrates underneath continuous graphene films for sensing protein A/G and goat anti-mouse immunoglobulin G bilayers [118]. Zhu et al. applied similar structures for low-molecular-weight analyte detection [119]. Specifically, very low concentrations of glucose (200 pM) were detected using suspended graphene over a very narrow (10 nm) gap between Au antennas. Lee et al. uncovered the two-stage coupling scheme for this type of structure between graphene film and Au nanoribbon arrays underneath (Fig. 7f) [107]. By combining the hybrid structure with a spacer and reflector, the absorption of the hybrid structure reached 94%, and the sensitivity towards ultrathin silk film (~ 2.4 nm) detection was enhanced by an order of magnitude.

Additionally, graphene-on-metal SPR prisms have been widely applied for bio-applications, such as detecting DNA and antibodies [120–122]. When analytes are attached onto the surface of graphene, the refractive index changes and the shift can be detected by the resonance angle from a prism. Mono- or bi-layer graphene can help enhance the sensitivity of conventional SPR prism coupled metal sensors.

### Chemical sensors

**Fig. 8:**
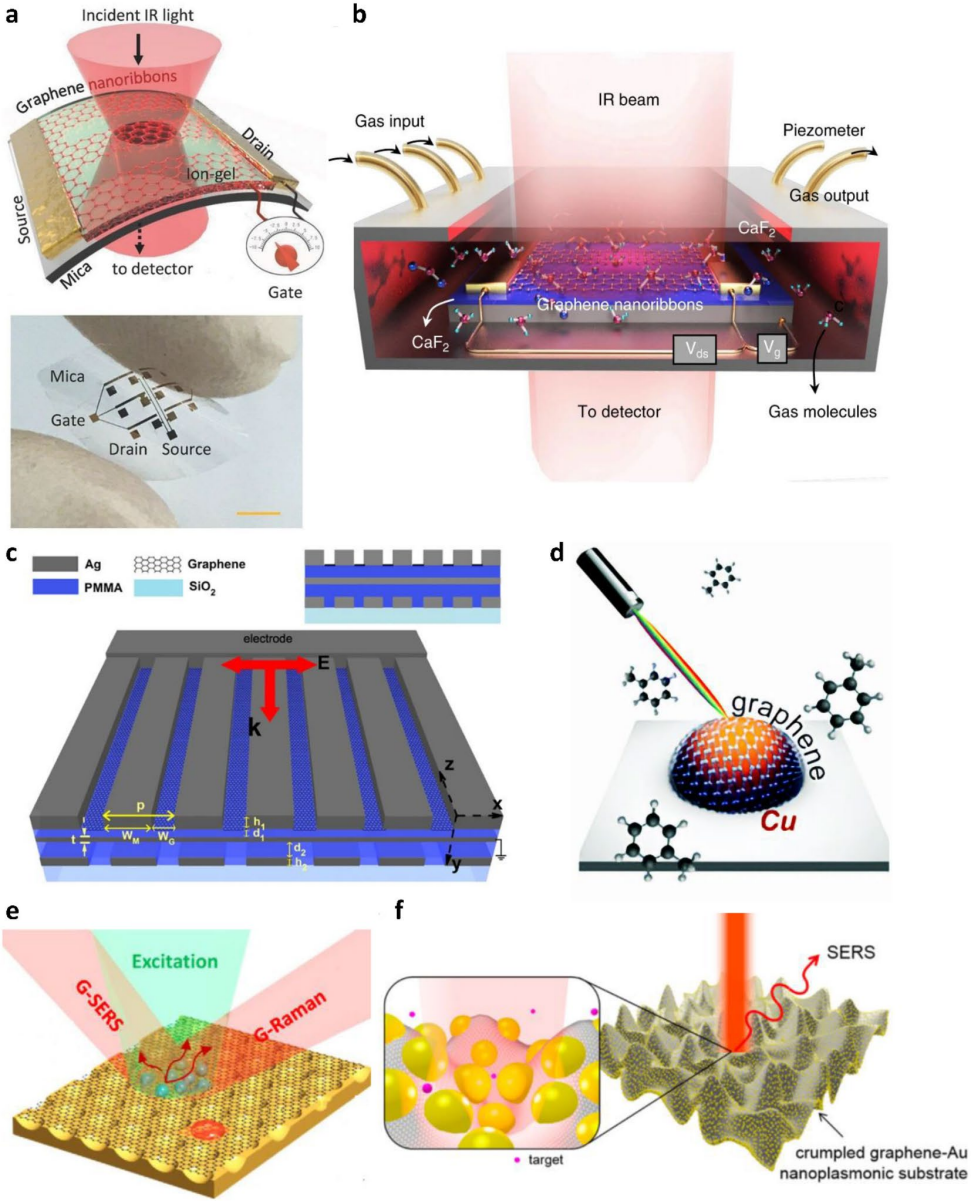
Graphene based plasmonic sensors for chemical sensing. **a** Plasmon excitation and detection with flexible graphene-mica plasmonic device for ion gel. Reproduced with permission from [14]. Copyright 2018 The Authors. Published by WILEY-VCH Verlag GmbH & Co. KGaA, Weinheim. **b** Graphene nanoribbon plasmonic gas sensor. A metal gas chamber with a piezometer was used for the precise control of gas parameters. Reprinted under the terms of the Creative Commons License from [15]. Copyright 2019 The Authors. Published by Springer Nature. **c** Ag nanoribbon-graphene hybrid structure on a PMMA/Ag/PMMA substrate for gas sensing. Reprinted under the terms of the Creative Commons License from [124]. Copyright 2016 The Authors. Published by Springer Nature. **d** Schematic illustration of local plasmonic resonance sensor based on graphene encapsulated copper nano-island. Republished with permission of Royal Society of Chemistry from [118]. Copyright 2020; permission conveyed through Copyright Clearance Center, Inc. **e** SERS sensor based on graphene-Au nanovoids. Reproduced with permission from [132]. Copyright 2013 American Chemical Society. **f** Schematic illustration of 3D crumpled graphene-Au NPs hybrid sensor. Reprinted with permission from [72]. Copyright 2015 American Chemical Society

The detection and tracking of small molecules in gas or liquid have attracted significant interest for monitoring health conditions, diagnosing diseases, or assessing hazards in food safety applications. Farmer et al. used graphene nanoribbon structures to detect the vibrational footprints in the extinction spectra of small organic molecule perylene-3,4,9,10-tetracarboxylic dianhydride as well as vapor-phase acetone and hexane [13]. When target molecules were attached onto graphene nanoribbons, much stronger absorption vibrational modes were observed in the extinction spectra of the graphene nanoribbons compared with graphene sensors without such plasmonic enhancement. Similarly, Hu et al. developed a flexible plasmonic ion-gel sensor based on graphene nanoribbons on mica substrates (Fig. 8a) [14]. The vibrational modes of ion-gel were detected from the extinction spectra of graphene plasmons. The authors additionally demonstrated that the performance of their devices, including resonance frequency, extinction intensity, quality factor, and graphene plasmon lifetime, was bending-insensitive. Later, the same group published another work on graphene nanoribbon-based plasmonic gas sensors (Fig. 8b) [15]. These sensors could detect and differentiate rotational-vibrational modes of toxic gas molecules SO_2_, NO_2_, N_2_O, and NO in real time. Due to the strong confinement of graphene plasmons and high adsorption of gas molecules on graphene, the graphene sensor’s detection limit was as low as 800 ppm. Bareza et al. further enhanced the adsorption of a target gas molecule, CO_2_, to lower the detection limit of their graphene-based plasmonic gas sensors by depositing an ultrathin layer of polyethylenimine (PEI) on top of graphene nanoribbons [16]. Because PEI is chemically reactive to CO_2_, the PEI-graphene hybrid structures were therefore able to detect CO_2_ fingerprintsat concentrations as low as 390 ppm from graphene extinction spectra. Further studies suggest that graphene SPR sensors combined with metal nanoantennas [123] or metal nanogratings (Fig. 8c) [124] can be used for detecting fingerprints of ambient gases and water vapor in graphene transmission spectra with enhanced sensitivity. Recently, Di Bernardo et al. developed a graphene-metal SPR sensor by using abundant and affordable Cu, underneath graphene (Fig. 8d) [125]. Graphene can be synthesized directly on Cu and protect the underlying Cu nano-islands from oxidation, resulting in devices with stability. Toluene was found to change the extinction and the resonance wavelength of the graphene plasmon absorption spectra. The resulting hybrid sensor was able to detect 1% toluene gas with 0.2 nm of resonance wavelength shift.

Plasmons in graphene and graphene-based hybrid materials have also been applied for sensing applications through another widely investigated technique, SERS. Conventionally, noble metal nanostructure arrays are used in SERS sensors for detecting molecules through two different mechanisms, EM and CM. EM is based on highly localized, confined electromagnetic field between adjacent plasmonic nanostructures while CM originates from charge transfers between sensing materials and target molecules [126]. Graphene has distinct advantages when compared to metal NPs as a material for SERS sensing. First, graphene promotes the attachment of target molecules physically or chemically through π-π interactions. Second, graphene reduces the FL background of SERS signals via the FL quenching effect. Ling et al. reported that graphene enhanced the Raman signals of adsorbed probe molecules by CM [54]. Analyte materials, including Pc, R6G, PPP, and CV were deposited on a graphene surface, and the resulting graphene plasmons exhibited Raman enhancements by a factor of 2–17 times. The same authors additionally reported more systematic studies for understanding the enhancement mechanisms of graphene SERS [127, 128]. However, graphene SERS have some intrinsic weaknesses, such as low light absorption and insufficient Raman peak characteristics [129–131].

Noble metal NPs combined with graphene can produce nanostructures suitable for SERS with enhanced light absorption and allow for more effective plasmonic sensing of molecules. Additionally, graphene can provide a flat surface for depositing uniform layers of metal NPs and protect the NPs from oxidation or corrosion. Zhu et al. reported graphene transferred onto a Au nanovoid array (Fig. 8e) [132]. They found that the coupling of graphene with metal voids caused significant frequency shifts of the plasmonic resonances of metal nanostructure, and a 30% enhancement of light absorption was achieved with such hybrid structures. As a result, Raman spectra of graphene were enhanced by ~ 700 times, and Raman spectra of R6G dye molecules were enhanced by ~ 1000 times. Losurdo et al. combined graphene with GaNPs and investigated their SERS effect using R6G dyes [133]. They compared two different structural configurations of graphene and Ga, Ga-on-graphene and Ga-under-graphene. Ga-under-graphene showed a higher enhancement factor of 50 with reduced background signals, which was attributed to the strong FL quenching effect of graphene and the highly ordered target molecules formed at the graphene surface. Leem et al. introduced 3D structures to graphene-metal hybrid systems and demonstrated further improved SERS enhancement factor (Fig. 8f) [72]. The 3D structure not only increased the density of hot spots, but also reduced the distances between NPs, leading to higher localized field enhancement. The sensitivity of the 3D Au/graphene hybrid structure was an order of magnitude higher when 4-mercaptiphenol (4-MPH) was used as an analyte, compared to a flat control sample.

Metal/graphene/metal hybrid structures can also serve as SERS substrates with high sensitivity. Nguyen et al. adopted graphene/Au film/Au nanorod hybrid structures for detecting pesticides including azinphos-methyl, carbaryl, and phosmet with detection limits of 5, 5, and 9 ppm, respectively [134]. Dong et al. fabricated flexible AuNPs/graphene/Au NPs sandwich structures as SERS substrates with R6G as the target analyte [80]. Their sandwich structure exhibited extremely low detection limits, down to 10^− 9^ M. These sensors were additionally applied for more practical chemical detection, such as detecting thiram from orange peels.

Graphene-based hybrid materials can also employs non-metallic structures. Fei et al. added dielectric PS microparticles to a graphene-based field-effect transistor for sensing noxious gas NO_2_ with enhanced SERS signals [135]. The detection limit was 45 ppb under dark conditions, but was reduced significantly to 0.5 ppb when the hybrid structure was illuminated with a near-IR laser. This enhancement was attributed to charge transfer between the PS beads and the graphene as well as the propagation of SPP waves.

### Graphene plasmonic optical sensors (photodetectors)

Although graphene exhibits broad-band optical absorption from ultraviolet to THz wavelengths, its low absorption and short excited carrier lifetime limit its application for photodetection. Plasmonic effects can largely enhance the light absorption, and graphene plasmons have much longer lifetimes. By utilizing plasmonic effects, the performance of graphene-based optical sensors can therefore be significantly improved. However, graphene SPPs cannot be directly excited by incident light because of the mismatch between graphene plasmon momentum and light momentum. To resolve this, researchers have either employed graphene nanostructures (e.g., nanoribbons, nanoflakes, or nanodisks) or combined graphene with other plasmonic structures in order to effectively excite graphene plasmons.

**Fig. 9:**
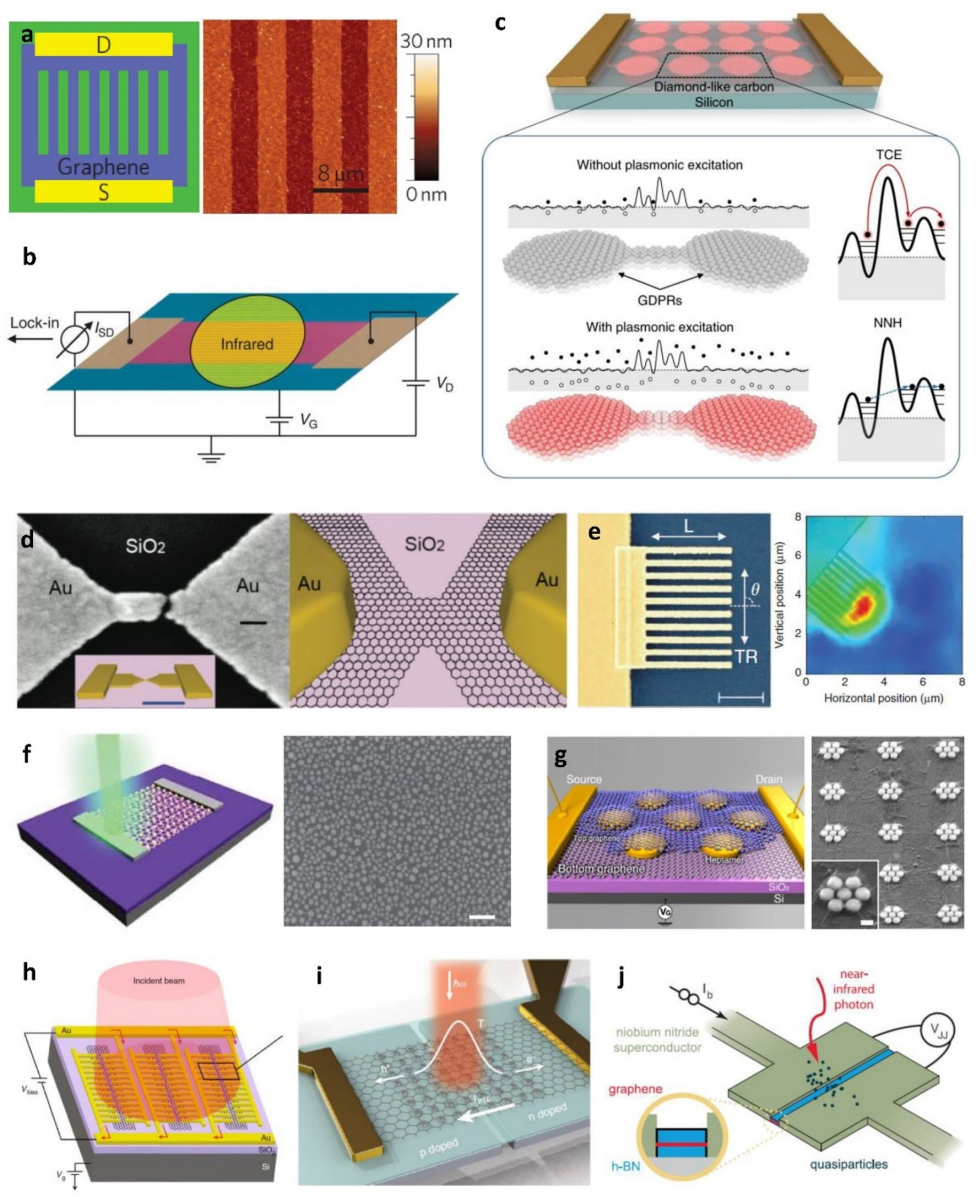
Graphene and graphene-based hybrid structures for photosensing. **a** Schematic illustration (left) and AFM image (right) of graphene micro-ribbon arrays for light detection in THz range. Reprinted by permission from Springer Nature Customer Service Centre GmbH: Springer Nature, Nature Nanotechnology, Graphene plasmonics for tunable terahertz metamaterials, Long Ju et al., Copyright 2011 [10]. **b** Schematic illustration of graphene nanoribbons for mid-IR light detection. Reprinted by permission from Springer Nature Customer Service Centre GmbH: Springer Nature, Nature Communications, Photocurrent in graphene harnessed by tunable intrinsic plasmons, Marcus Freitag et al., Copyright 2013 [17]. **c** GDPRs (red circles) connected by quasi-1D graphene nanoribbons (GNRs). Dashed lines represent the unperturbed chemical potential. Solid curves are the disorder potential. Filled and open circles refer to electrons and holes, respectively. The bottom right shows two charge carrier transport mechanisms: thermal-carrier excitation (TCE) transport and nearest-neighbor hopping (NNH) transport. Reprinted by permission from Springer Nature Customer Service Centre GmbH: Springer Nature, Nature Materials, Efficient electrical detection of mid-infrared graphene plasmons at room temperature, Qiushi Guo et al., Copyright 2018 [21]. **d** SEM image (left) and schematic illustration (right) of an Au nanogap antenna with graphene in the gap. Scale bar is 100 nm (5 μm for inset). Reprinted with permission from [136]. Copyright 2011 American Chemical Society. **e** Finger type Ti/Au plasmonic nanostructures on graphene. The right figure indicates the photovoltage map illuminated with 514 nm light with transverse polarization. Scale bar is 1 μm. Reprinted by permission from Springer Nature Customer Service Centre GmbH: Springer Nature, Nature Communications, Strong plasmonic enhancement of photovoltage in graphene, Echtermeyer et al., Copyright 2011 [137]. **f** Schematic illustration of graphene photodetector with AuNPs (left), and SEM image of AuNPs on a graphene surface (right). Scale bar is 100 nm. Reprinted by permission from Springer Nature Customer Service Centre GmbH: Springer Nature, Nature Communications, Plasmon resonance enhanced multicolour photodetection by graphene, Yuan Liu et al., Copyright 2011 [112]. **g** Schematic illustration of a single Au heptamer sandwiched between two monolayer graphene sheets (left) and SEM image of an Au heptamer (right). The scale bar in the inset of the right figure is 100 nm. Reproduced with permission from [138]. Copyright 2012 American Chemical Society. **h** Photodetector based on Au-patched graphene nano-stripes for utilizing maximum metal-graphene interfaces for enhanced photocurrent. Reprinted under the terms of the Creative Commons License from [142]. Copyright 2018 The Authors. Published by Springer Nature. **i** Schematic illustration of a graphene photodetector integrating both optical heating enhancement (*via* gap plasmonic structures) and electrical junction enhancement (via split gates). Reprinted under the terms of the Creative Commons License from [144]. Copyright 2020 The Authors. Published by Springer Nature. **j** Schematic illustration of a single photon detection device using a Josephson junction. From [147]. Reprinted with permission from AAAS

Ju et al. experimentally demonstrated light-plasmon coupling in graphene at room temperature using graphene micro-ribbon arrays on SiO_2_/Si substrates (Fig. 9a) [10]. The plasmonic resonances can be tuned in a broad range at THz frequency by changing the graphene ribbon width or electrostatic doping. Researchers have further studied the mechanisms of plasmonic photocurrent generation of graphene nanostructures in IR and THz range and demonstrated optical sensing applications. Freitag et al. applied periodic graphene nanoribbon arrays for mid-IR light detection (Fig. 9b) [17]. The graphene plasmons interacted with the phonons of SiO_2_ substrates, forming long-lived plasmon-phonon modes with narrow spectral widths. The increase in lattice temperature due to the photothermal effect of graphene plasmons in the nanoribbon was more than 4 times higher than that of continuous graphene sheets, and the polarization effect strengthened when the nanoribbon width decreased under 100 nm.  Badioli et al. further demonstrated that the mid-IR absorption of graphene micro-ribbons can be enhanced by coupling graphene plasmons either with bulk optical phonons, or with substrate surface optical phonons [18].

Plasmons in more complex graphene structures have also been investigated and more advanced functionalities have been demonstrated. Guo et al. employed graphene nanostructures with patterns, which consist of arrays of graphene-disk plasmonic resonators (GDPRs) connected by graphene nanoribbons as shown in Fig. 9c [21]. The GDPRs generated thermalized carriers by plasmonic resonance, and the graphene nanoribbons converted the thermal energies into detectable electrical signals. The device was operated at room temperature and exhibited subwavelength footprint, good photoresponsivity, and low noise-equivalent power, indicating potential for fundamental studies and practical applications.

On the other hand, graphene combined with other plasmonic materials for photodetection have been widely investigated for enhancing or controlling optical properties and photocurrent generations. Shi et al. demonstrated the photoresponse of a hybrid structure of Au nanogap antennas and graphene (Fig. 9d) [136]. As the gap size decreased from 100 nm to sub-10 nm, the photoresponse changed from antisymmetric thermal response to symmetric rectification response. The metal nanoantennas with sub-10 nm scale nanogap highly concentrated the incident light (near-IR) and largely enhanced the localized E-field at the gap. The device showed a high sensitivity towards the polarization of light (~ 99%), and the photocurrent was plasmonically enhanced by the factor of 2 to 100. Echtermeyer et al. combined graphene with different shapes of plasmonic metal nanostructures to form hybrid materials, one of which is illustrated in Fig. 9e [137]. Localized field enhancement and a p-n junction were generated when visible to near-IR light was illuminated at the graphene-metal interfaces. The hybrid structure of graphene with finger type metal nanostructures showed the best efficiency among different nanostructures with an enhancement factor of 20 when compared to plain graphene photodetectors. Liu et al. investigated hybrid structures of graphene with AuNPs (Fig. 9f) [112]. Their hybrid photodetectors showed an enhanced external quantum efficiency by 15-fold. Additionally, by tuning the shape, size, and density of the nanostructures, the sensor achieved good spectral selectivity, allowing it to detect different colors in the visible range. Fang et al. demonstrated a graphene–metal nanoantenna (Fano-resonant plasmonic clusters)–graphene sandwich structure exhibiting an 800% enhancement in photocurrent within the visible and near-IR regimes [138]. The metal antenna design is presented in Fig. 9g. The authors observed that the photocurrent was contributed both by direct plasmon excitations of graphene as well as by hot electrons generated in metal antennas.

Various strategies have been proposed and demonstrated for achieving even higher photoresponsivity. Nanofabrication strategies such as e-beam lithography can be used for creating metal nanostructures with precise control of antenna size and shape as well as the gap between adjacent antennas for stable and uniform photosensing performance. For nanorod arrays with a 60 nm nanogap, the photoresponsivity towards mid-IR light was enhanced by more than 200 times to 0.4 V/W compared to the responsivity of photodetectors without nanostructures (< 2 mV/W) [139]. By controlling nanoantenna geometries, graphene-based hybrid materials can interact with a broader range of incident light. For example, THz emission and detection was achieved by using graphene with Au nanogap antennas of 45 μm × 1 μm and an on-chip silicon lens [140]. The photodetector operated at ~ 2 THz and the maximum photoresponsivity achieved was 4.9 V/W. In addition to tailoring antenna geometries, introducing 3D structures to graphene-based photodetectors has been demonstrated to yield higher sensitivity. Kim et al. developed stretchable photodetectors with crumpled graphene/AuNPs hybrid structures. Their sensors could be stretched up to 300% and showed 12-fold enhancement in photoresponsivity (0.044 mA/W), when compared to flat graphene photosensors without AuNPs [141]. Cakmakyapan et al. developed a broadband detector consisting of graphene nano-strips with Au patches on top (Fig. 9 h) [142]. The close patterns enabled the graphene-based photosensor to exhibit high responsivity (0.6–11.5 A/W in 0.8–20 μm wavelength region) without sacrificing its ultrafast response speed (50 GHz). Shautsova et al. adopted asymmetric contacts to enhance the photosensitivity by 5 times with a fast response time of 2 ps due to strong photo-thermoelectric effect [143]. Wang et al. improved the sensing efficiency of a graphene-based photosensor by 25 times with two steps, light-to-heat and heat-to-electricity, by using plasmonic structure nanogaps and split-gates (Fig. 9i) [144]. Additionally, plasmonic metamaterials with different designs have been combined with graphene for detecting broadband wavelengths from visible to near-IR, with enhanced photoresponse and polarization-insensitive performance [145, 146]. Most recently, detection of a single near-IR photon has been achieved with graphene-based Josephson junctions (JJs) (Fig. 9j) [147].

### Other sensors

In addition to the aforementioned applications that have been widely investigated, plasmonic structures of graphene and graphene-integrated hybrid materials have additionally shown great potential for other sensing purposes. Dabidian et al. used graphene with a plasmonic metasurface layer to develop an interferometer for detecting nanoscale motions of reflecting objects [148]. The distance change between the reflecting object (mirror) and beam splitter (BS) in the Michelson interferometer setup led to a phase shift of the reflected mid-IR light. This phase shift was measured to determine the distance change (motion). Graphene has also been used in strain sensors by integrating with Au plasmonic nanogratings on flexible and stretchable substrates [149]. Applying mechanical strain changed the periodicity and intensity of the metal nanogratings on polydimethylsiloxane (PDMS) substrates, thereby inducing a Raman shift in spectrum. Graphene plasmons can also be applied for sensing polarization. Previous studies have demonstrated that graphene plasmonic structures can be sensitive to polarization of incident light. [11, 17, 18, 21, 116, 124, 136, 138, 139, 143, 148, 150] However, those structures usually showed limited polarization ratios (PRs) under 10.  Wei et al. demonstrated a nanoantenna-mediated graphene polarimeter with an extremely broad PR range [151]. By tuning the orientations of the metal nanoantennas, full coverage of PR values was achieved (1 → ∞/− ∞ → − 1), with minimum perturbation in polarization angle of 0.02^o^/Hz detected for mid-IR incident light. Lastly, theoretical studies have suggested that graphene plasmonic structures can be used for pressure and mass sensing [152, 153].

## Conclusions and outlook

The past decade has witnessed a boost of fundamental studies of graphene plasmons as well as their applications in biosensors, chemical sensors, photodetectors, and other fields. Graphene enables high electromagnetic field confinements with low losses and long lifetimes. The plasmonic properties of graphene can be easily tuned by electrical gating or chemical doping. Moreover, graphene plasmons are in the wavelength range (THz and mid-IR) that other plasmonic materials cannot reach. These distinctive optical properties of graphene provide a foundation for various plasmonic sensing applications. However, in order to access graphene plasmons, the momentum mismatch between graphene plasmons and incident light must first be overcome. Researchers have employed different approaches for exciting plasmons in graphene, including patterning graphene into micro/nano patterned structures, introducing defects and curvatures, applying selective doping, or forming Moiré superlattices. Other plasmonic nanomaterials, including NPs, nano gratings, and metamaterials, have also been adopted and integrated with graphene for tuning plasmonic properties and enhancing optical absorption.

Graphene additionally shows potential for plasmonic applications with ultrahigh sensitivities towards very low concentrations of analytes and even label-free single molecule detection. Beyond detecting materials, surface plasmon of graphene can also be used for detecting property or structural changes within films, which may largely broaden its application in monitoring, diagnosing, and characterization. In addition, due to its high carrier mobility (theoretically > 2.5 × 10^5^ cm^2^/Vs) and high Fermi velocity only two orders of magnitude lower than the speed of light [154], graphene shows promise for ultra-high speed detectors. Future perspectives also include extensions of graphene plasmonics to include other 2D materials (such as transition metal dichalcogenides (TMD), hBN, and phosphorene) and vdW heterostructures of graphene with other 2D materials. Beyond monolayer graphene, bilayer and trilayer graphene offer many potential avenues for future investigations. The stacking of graphene layers may improve absorption while simultaneously offering more options for tuning plasmonic properties [155–157]. It has been found that bilayer graphene with Bernal stacking exhibits greater plasmon confinement with tunable electrostatic gating enabling plasmon shut off [158, 159]. Additionally, applying Moiré physics with twisted bilayer graphene (or other vdW heterostructures) may provide further interesting results. Interlayer twisting provides an additional degree of freedom for bilayer systems and can create strong correlated quantum states, thereby affecting photonic behaviors such as SPPs [40, 156, 157]. Investigating the Moire physics in twisted vdW 2D layered materials is relatively new, and the effects on plasmonic properties have not yet been thoroughly explored, with potential to inspire next generation nanophotonic devices. Additionally, quasiparticles formed by interactions with graphene plasmons, including plasmarons, bound states of charge carriers with graphene plasmons[155, 160, 161] and plexcitons, polaritonic modes resulted from plasmon-exciton coupling [162, 163] all offer additional avenues of study. Investigations and applications of these interactions are still in their infancy and understanding these interactions may help further enhance the performance of plasmonic sensors.

Although graphene plasmons have demonstrated great potential for various sensing applications, challenges still remain towards commercialization. Commercialization will not only require consistent device performance, but also demand scalable, reliable, and consistent fabrication processes with low costs. Currently, most graphene plasmonic sensor fabrication requires complex processes with expensive facilities. Moreover, reproducibility and quality-control in mass-production of graphene-based plasmonic sensors has not yet been fully addressed, as most lab-scale fabrications only yield one or a few devices at a time. In terms of device performance, synthesizing high quality graphene with single crystallinity and fewer defects will be the highest priority for graphene-based plasmonic sensors. Specifically, graphene with improved quality will have greater mobility and longer lifetime, thereby enhancing absorption, light-matter interactions, efficiency, spectral resolution, and sensitivity. From this point of view, batch production of high quality graphene via CVD [164–168], may serve as a scalable platform for producing high-performance graphene-based plasmonic sensors. In addition, many nano/micro fabrication processes produce significant chemical waste, requiring development of sustainable fabrication processes for commercialization to occur. Lastly, packaging and integration into existing platforms such as integrated device platforms pose additional challenges for commercialization as most plasmonic sensors require external light sources for exciting plasmons with background light blocked.

## Data Availability

Not applicable.

## Abbreviations

2D: Two-dimensional
IR: Infrared
THz: Terahertz
SPP: Surface plasmon polariton
AFM: Atomic force microscopy
E-field: Electric field
SPR: Surface plasmon resonance
E_z_: Out-of-plane E-field
vdW: Van der Waals
hBN: Hexagonal boron nitride
SPPP: Surface plasmon–phonon polariton
FL: Fluorescence
SERS: Surface enhanced Raman scattering
R6G: Rhodamine 6G
PPP: Protoporphyrin IX
CM: Chemical mechanism
HOMO: Highest occupied molecular orbital
LUMO: Lowest unoccupied molecular orbital
Pc: Phthalocyanine
CV: Crystal violet
EM: Electromagnetic mechanism
NPs: Nanoparticles
CVD: Chemical vapor deposition
3D: Three-dimensional
PMMA: Poly(methyl methacrylate)
PVP: Poly(vinylpyrrolidone)
PE: Polyethylene
PEO: Polyethylene oxide
FP: Fabry–Perot
PBS: Phosphate buffer saline
DA: Dopamine
GSH: Glutathione
DTSP: 3,30-Dithiodipropionic acid di(N-succinimidyl ester)
PS: Polystyrene
UV–vis: Ultraviolet–visible
CoPc: Cobalt phthalocyanine
TNAs: Triangular nanoarrays
E_F_: Fermi energy
PABA: Para-aminobenzoic acid
CSRR: Complementary split ring resonator
PTCDA: Perylene-3,4,9,10-tetracarboxylic dianhydride
PEI: Polyethylenimine
4-MPH: 4-Mercaptiphenol
GDPR: Graphene-disk plasmonic resonator
JJ: Josephson junction
NNH: Nearest-neighbor hopping
PDMS: Polydimethylsiloxane
PR: Polarization ratio
TMD: Transition metal dichalcogenides
